# Depletion of davemaoite in Earth’s ambient lower mantle

**DOI:** 10.1038/s41467-026-76943-0

**Published:** 2026-08-15

**Authors:** Ming Hao, Motohiko Murakami, Pinku Saha, Nicolas Guignot

**Affiliations:** 1 Department of Earth and Planetary Sciences, Institute of Geochemistry and Petrology, ETH Zürich, Zürich, Switzerland; 2 Soleil Synchrotron, French National Centre for Scientific Research, Saint-Aubin, France

**Keywords:** Mineralogy, Seismology, Geophysics

## Abstract

The mineralogy of the Earth’s lower mantle is fundamental to understanding the planet’s formation, composition, evolution, and dynamics. However, uncertainties in the elastic properties and phase transitions of CaSiO₃ davemaoite—the third most abundant phase in a pyrolitic lower mantle—have hindered quantitative links between seismic observations and lower-mantle mineralogy. Here we combine Brillouin spectroscopy with resistively heated diamond anvil cell experiments to measure the sound velocities of CaSiO₃ davemaoite up to 80 GPa and 973 K. We show that the tetragonal–cubic phase transition occurs below 900 K throughout the entire lower mantle. Fitting the thermoelastic properties of cubic davemaoite reveals substantially reduced shear-wave velocities at deep lower-mantle conditions. Because of these intrinsically slow velocities, increasing bridgmanite abundance alone cannot reconcile seismic observations for a normal lower mantle containing even small amount of davemaoite. Our results therefore suggest that davemaoite is depleted in the ambient lower mantle but concentrated within LLSVPs, likely as a consequence of late-stage crystallization during magma-ocean differentiation.

## Abstract (web summary)

Davemaoite remains cubic throughout most of the lower mantle and exhibits intrinsically low shear-wave velocities. Seismic constraints indicate that it is depleted in the ambient lower mantle but enriched within LLSVPs, likely as a result of late-stage crystallization during magma-ocean differentiation.

## Introduction

The Earth’s lower mantle, extending from ~660 to 2900 km depth (~25–135 GPa), constitutes more than half of the planet’s volume and exerts a first-order control on Earth’s thermal, chemical, and dynamical evolution. Accurate knowledge of the physical and chemical properties of lower-mantle materials is therefore essential for interpreting seismic observations and for constraining models of deep-Earth structure and planetary evolution. At present, our understanding of the lower mantle relies largely on seismic observations, such as one-dimensional and regional seismic velocity models, which in turn require accurate constraints on the elastic properties of relevant materials under high-pressure and high-temperature conditions.

Previous phase-equilibrium experiments^1,2^, indicate that davemaoite (CaSiO_3_) is the third most abundant mineral phase in the Earth’s lower mantle. At low temperatures, davemaoite adopts a tetragonal structure (space group *I4/mcm*), which transforms into a cubic structure (space group ${Pm}\bar{3}m$) with increasing temperature. Both experimental and theoretical studies^3–8^ demonstrate that the elastic properties—particularly the shear modulus (*G*)—differ substantially between the tetragonal and cubic phases. Consequently, accurately constraining the phase boundary between tetragonal and cubic davemaoite under lower-mantle pressure–temperature conditions is critical for interpreting seismic observations of the lower mantle.

Despite its importance, the phase boundary reported by different studies remains controversial. X-ray diffraction (XRD) studies that infer the transition from peak splitting and broadening suggest that the cubic phase is stable at temperatures well below the mantle geotherm throughout the lower mantle^9,10^, even in the coldest subducting slabs. In contrast, theoretical calculations predict a much steeper Clapeyron slope, allowing the phase boundary to intersect the mantle geotherm near the base of the lower mantle^5,7^. These discrepancies may reflect limitations in resolving closely spaced diffraction peaks in XRD experiments^9^, as well as uncertainties associated with anharmonic and quantum effects in theoretical calculations^11^. Experimental constraints using other methods instead of XRD are therefore needed to clarify the discrepancy. Notably, low-pressure ultrasonic measurements have shown that sound velocities change continuously across the phase transition^3,4^, highlighting high-temperature elasticity measurements as a sensitive probe of the structural transition.

Large discrepancies among published studies also persist in the absolute elastic properties of davemaoite. Existing experimental studies report significantly lower shear-wave velocities than theoretical predictions for both high-pressure tetragonal davemaoite at room temperature and cubic davemaoite at elevated temperatures^3,6,7,11,12^. However, due to experimental challenges, elasticity data for cubic davemaoite at true lower-mantle pressures remain scarce, and most mineralogical models either neglect davemaoite^13,14^ or rely on extrapolations from low-pressure measurements^15,16^. Reliable sound velocity data for cubic davemaoite under true lower-mantle conditions are therefore essential for accurately assessing lower-mantle mineralogy.

In this study, we investigate the phase transition and elastic properties of davemaoite under lower-mantle conditions by measuring shear-wave velocities using Brillouin spectroscopy up to 80 GPa and 973 K. By combining our high-pressure, high-temperature data with existing low-pressure ultrasonic measurements, we precisely constrain the phase boundary between the tetragonal and cubic structures. Furthermore, by integrating our velocity results with previous volume measurements, we derive updated thermoelastic parameters for davemaoite applicable to lower-mantle conditions. Our results provide new constraints on the role of davemaoite in shaping seismic heterogeneity and mineralogy in the Earth’s lower mantle.

## Results and discussion

### Phase transition of davemaoite

**Fig. 1:**
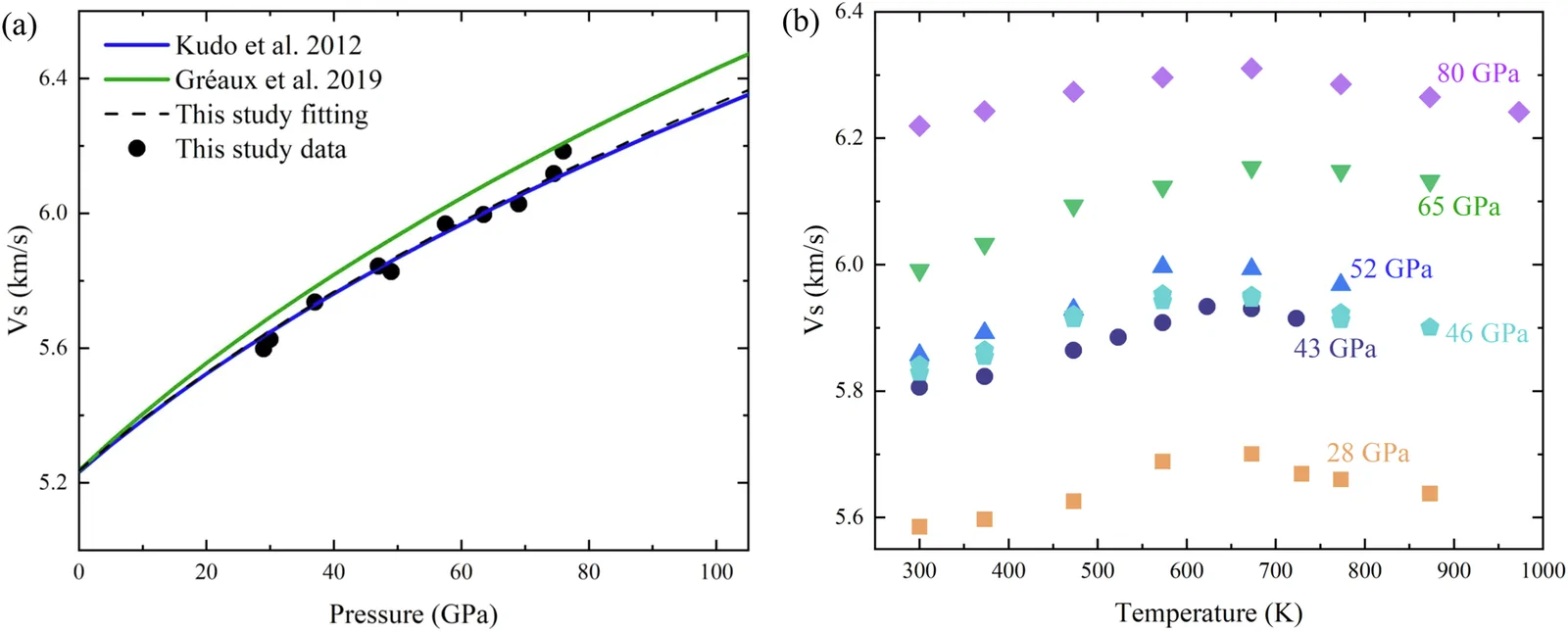
Shear wave velocities of tetragonal and cubic davemaoite. **a** Room-temperature (300 K) shear-wave velocities compared with previous measurements^3,12^. For consistency, the fitting equations used here are the same as those adopted in previous studies^3,12^, which differ slightly from the equations described in the “Methods” section. **b** Shear-wave velocities measured at high temperatures. Small pressure variations during individual heating cycles were corrected using the pressure dependence derived from fitted elastic parameters for tetragonal^12^ and cubic davemaoite. Uncertainties are provided in Supplementary Table 1. (Orange squares, 28 GPa; dark blue circles, 43 GPa; cyan pentagons, 46 GPa; blue upward triangles, 52 GPa; green downward triangles, 65 GPa; and purple diamonds, 80 GPa.).

As shown in Fig. 1a, the measured room-temperature shear-wave velocities (*Vs*) of davemaoite are consistent with previous studies^3,12^, demonstrating the reliability of our measurements. During each heating cycle, the pressure varied slightly—either increasing or decreasing—depending on the anvil seat material (Si₃N₄ or ZrO₂; Supplementary Table 1). To isolate the intrinsic temperature effects on sound velocity at fixed pressure, all high-temperature velocity data were corrected for pressure variations using the pressure dependence of the elastic properties of tetragonal^12^ and cubic davemaoite (Supplementary Table 2). After correction, the temperature dependence of *Vs* exhibits a consistent and systematic trend across all experiments (Fig. 1b), regardless of whether pressure increased or decreased during heating. Specifically, *Vs* initially increases with temperature, reflecting the progressive reduction of tetragonal distortion and the gradual evolution of the structure toward cubic symmetry. Once temperatures exceed ~600-700 K, *Vs* decreases monotonically with further heating. Different from the significant softening of CaTiO_3_ system during phase transition^4,17^ and the recent theoretical calculations for CaSiO_3_^7^, we did not observe any softening for CaSiO_3_. This behavior closely matches trends observed in previous ultrasonic measurements^3,4^, confirming that the temperature at which *Vs* changes from a positive to a negative temperature dependence provides a robust and precise constraint on the tetragonal–cubic phase boundary of davemaoite. The elastic softening observed in the low-temperature tetragonal phase of davemaoite may result from additional octahedral rotations associated with its asymmetric distortion^5^ or from a temperature-activated twin-domain-wall process^17^. As temperature increases toward the phase boundary, the tetragonal distortion progressively diminishes and the structure gradually approaches cubic symmetry^9^. Correspondingly, the sound velocity increases continuously with temperature until the phase transition is reached.

**Fig. 2:**
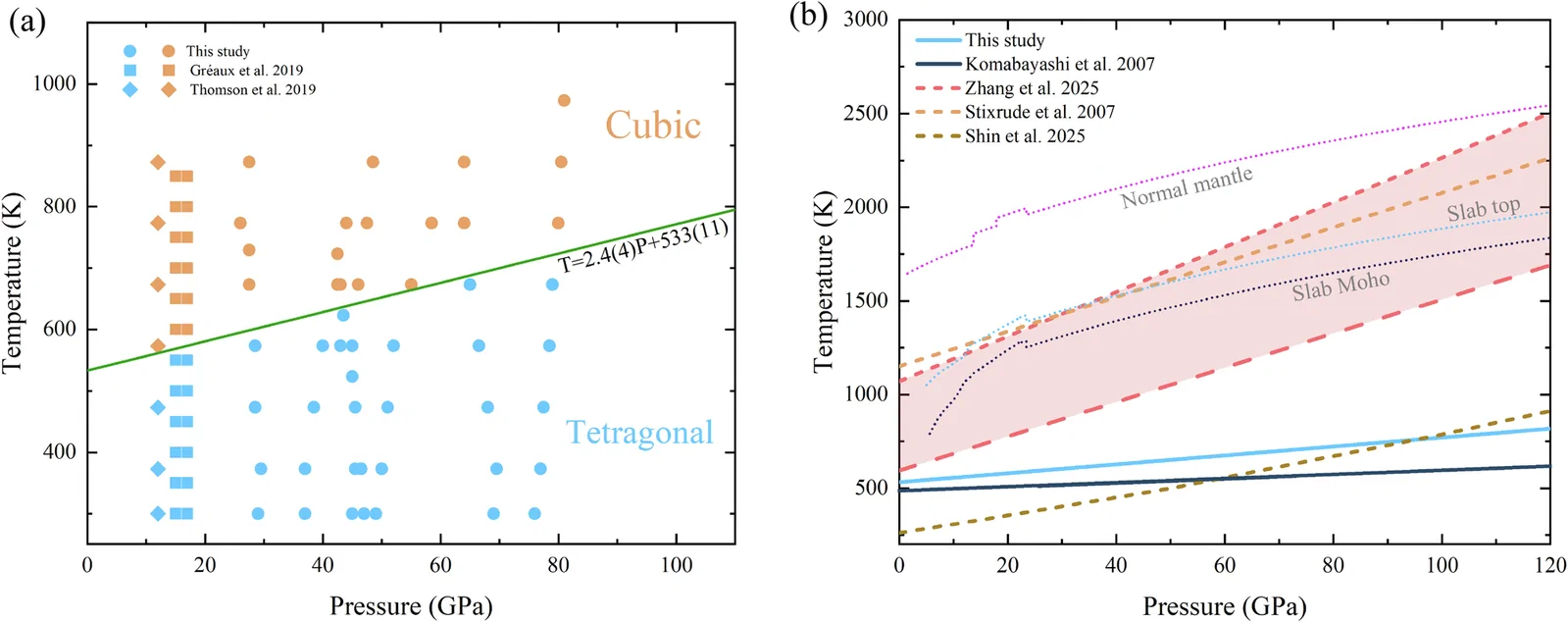
Phase boundary between tetragonal and cubic davemaoite. **a** Phase boundary defined by shear-wave velocity measurements. The orange and blue colors represent cubic and tetragonal structures, respectively. **b** Comparison of phase boundaries reported in previous studies^5,7,9–11^. Solid and dashed lines denote experimental and theoretical results, respectively. Sun et al. ^10^ did not directly constrain the phase boundary but demonstrated that davemaoite is cubic above 1200 K across the entire lower-mantle pressure range. The slab geotherm shown corresponds to the coldest subduction model from Shirey et al. ^18^, extrapolated into the lower mantle along a reference mantle geotherm^27^.

As shown in Fig. 2a, combining our results with these ultrasonic data, the tetragonal–cubic phase boundary of davemaoite is constrained to 533(11) K at 0 GPa with a Clapeyron slope of 2.4(4) K GPa⁻¹. Fig. 2b summarizes phase boundaries reported in previous experimental and theoretical studies^5,7,9–11^. Our experimentally determined phase boundary is broadly consistent with prior X-ray diffraction measurements^9^, although it occurs at slightly higher temperatures. In contrast to early theoretical predictions^5,7^, which suggested that the phase transition could intersect mantle geotherms near the base of the lower mantle, all available experimental constraints indicate that the tetragonal-to-cubic transition temperature remains below ~1200 K throughout the entire lower mantle. A recent theoretical study^11^ proposed that earlier discrepancies arose from inaccurate treatments of anharmonic and quantum effects; when these effects are more accurately accounted for, the revised theoretical predictions match experimental results better, albeit with a modestly steeper Clapeyron slope (Fig. 2b). Based on the refined phase boundary for pure CaSiO₃ davemaoite established here, the tetragonal phase is not stable under lower-mantle conditions, even within the coldest subducting slabs^18^, which is consistent with previous X-ray measurements^9,10^.

Large Low Shear Velocity Provinces (LLSVPs) are vast anomalous regions in the lowermost mantle beneath Africa and the Pacific Ocean, characterized by shear-wave velocity reductions of ~2–5% relative to the surrounding mantle^19,20^. These structures are widely interpreted as thermochemical heterogeneities, potentially derived from subducted materials or preserved primordial components associated with a basal magma ocean^6,21^. Zhang et al. ^7^ proposed that softening due to tetragonal–cubic phase transition in davemaoite could account for the seismic signature of LLSVPs, assuming that the softening temperature regions (500–800 K higher than phase boundary) intersects mantle geotherms near the base of the lower mantle. However, the experimentally constrained phase boundary established in this study indicates a much lower transition temperature (~860 K at the base of the lower mantle). Even considering the width of the softening region predicted by theoretical calculations^7^, the tetragonal davemaoite—and any associated elastic softening region, if present—is unlikely to be stable within the lower mantle. Consequently, the tetragonal–cubic phase transition of davemaoite cannot contribute to the observed shear-wave velocity reductions associated with LLSVPs, effectively ruling out this mechanism as an explanation for their seismic anomalies.

### Elastic properties of cubic davemaoite

**Fig. 3:**
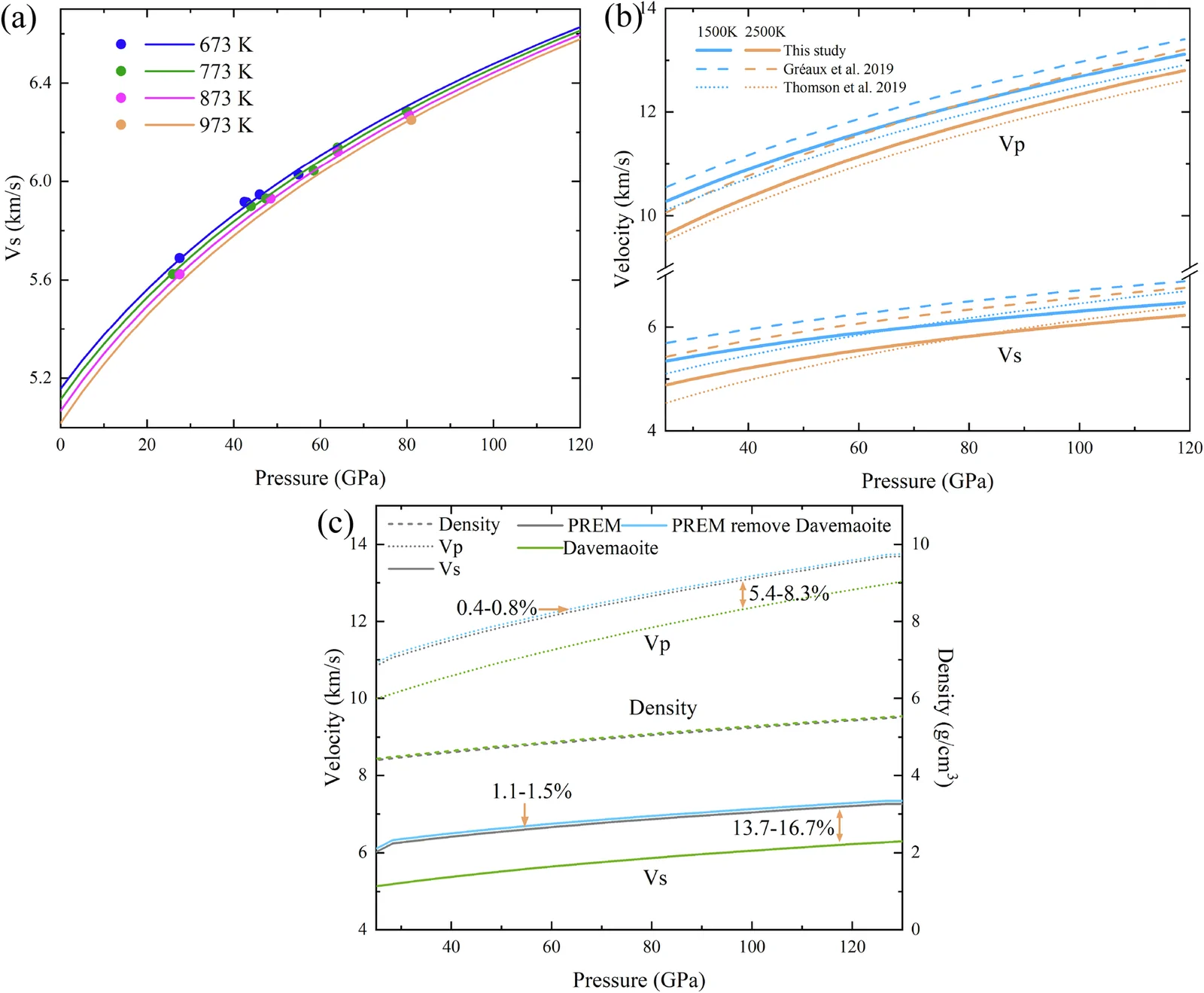
High-temperature elastic properties of cubic davemaoite. **a** Fits to the elastic data of cubic davemaoite obtained in this study. Uncertainties and velocities at additional temperatures are provided in Supplementary Table 1. **b** Comparison of experimentally measured sound velocities of cubic davemaoite from this study and previous measurements^3,4^. **c** Predicted seismic velocities and density of davemaoite along an adiabatic mantle geotherm^27^. Blue curves show the calculated seismic velocities of a lower-mantle assemblage after removal of 7 vol.% davemaoite.

By combining the *Vs* measurements of cubic davemaoite obtained in this study with all available volume data from previous experiments^3,4,10,22–24^ (Supplementary Data 1), we constrained the elastic properties of cubic davemaoite using a third-order Eulerian finite-strain equation of state (EOS; see “Methods”)^25,26^. Fixing the ambient-pressure volume to *V*_*0*_ = 45.58 Å^3^ based on prior experimental studies^3,23^, the fitting yields *K*_*t0*_ = 243(4) GPa, *K*_*t0*_*’* = 4.09(8), *K*_*S0*_ = 245(4) GPa, *K*_*S0*_*’* = 4.05(8), *G*_*0*_ = 118(2) GPa, *G*_*0*_*’* = 1.44(2), *θ*_*0*_ = 1450(300) K, *γ*_*0*_ = 2.0(2), *q*_*0*_ = 1.1(4), and *η*_*0*_ = 2.8(13) (Fig. 3a). Fig. 3b compares sound velocities of CaSiO₃ davemaoite under high-pressure and high-temperature conditions calculated using experimentally derived parameters from different studies^3,4^ (Supplementary Table 2). Relative to previous ultrasonic measurements, the present study yields intermediate values of *G*_*0*_ and temperature dependence, but a smaller pressure derivative *G*_*0*_*’*. The discrepancies in *G*_*0*_ and its temperature dependence reported in previous ultrasonic studies^3,4^ may reflect the challenges associated with accurately constraining sample dimensions, as well as potential deformation during the glass-to-crystal transition and subsequent heating. The larger *G*_*0*_*’* reported in earlier ultrasonic studies are likely due to the limited pressure range. As a consequence of the reduced pressure dependence determined here, shear-wave velocities of davemaoite in the deep lower mantle are substantially lower than extrapolations based on low-pressure ultrasonic data.

Fig. 3c shows the calculated sound velocities and density of davemaoite along an adiabatic mantle geotherm^27^, compared with the Preliminary Reference Earth Model (PREM)^28^. Throughout the entire lower mantle, davemaoite exhibits significantly lower seismic velocities than the ambient mantle, while its density is slightly higher. Notably, the reduction in *Vs* is substantially larger (−13.7 to −16.7%) than that in *Vp* (−5.4 to −8.3%). Despite constituting only ~7 vol.% of a pyrolitic lower mantle^1,13^, davemaoite exerts a disproportionate influence on seismic properties owing to its strongly reduced velocities. For example, mineralogical models of the lower mantle that neglect davemaoite should predict *Vp* and *Vs* that exceed PREM by ~0.4–0.8% and ~1.1–1.5%, respectively (Fig. 3c).

### Lower mantle mineralogy

Phase-equilibrium experiments indicate that the Earth’s lower mantle is dominated by three major mineral phases: bridgmanite, ferropericlase, and davemaoite^1,2^. Accurately determining lower-mantle mineral proportions is essential not only for constraining Earth’s bulk composition but also for understanding mantle convection and long-term planetary evolution. By combining seismic observations with mineral elastic properties, the volume proportions of these phases can, in principle, be constrained. However, large uncertainties persist owing to the lack of reliable elastic data for cubic davemaoite under lower-mantle conditions and to discrepancies among reported elastic properties of bridgmanite^13–16,29^. As a result, estimates of bridgmanite abundance in the lower mantle range widely, from ~75 vol.% to more than 90 vol.%^14–16,29^.

**Fig. 4:**
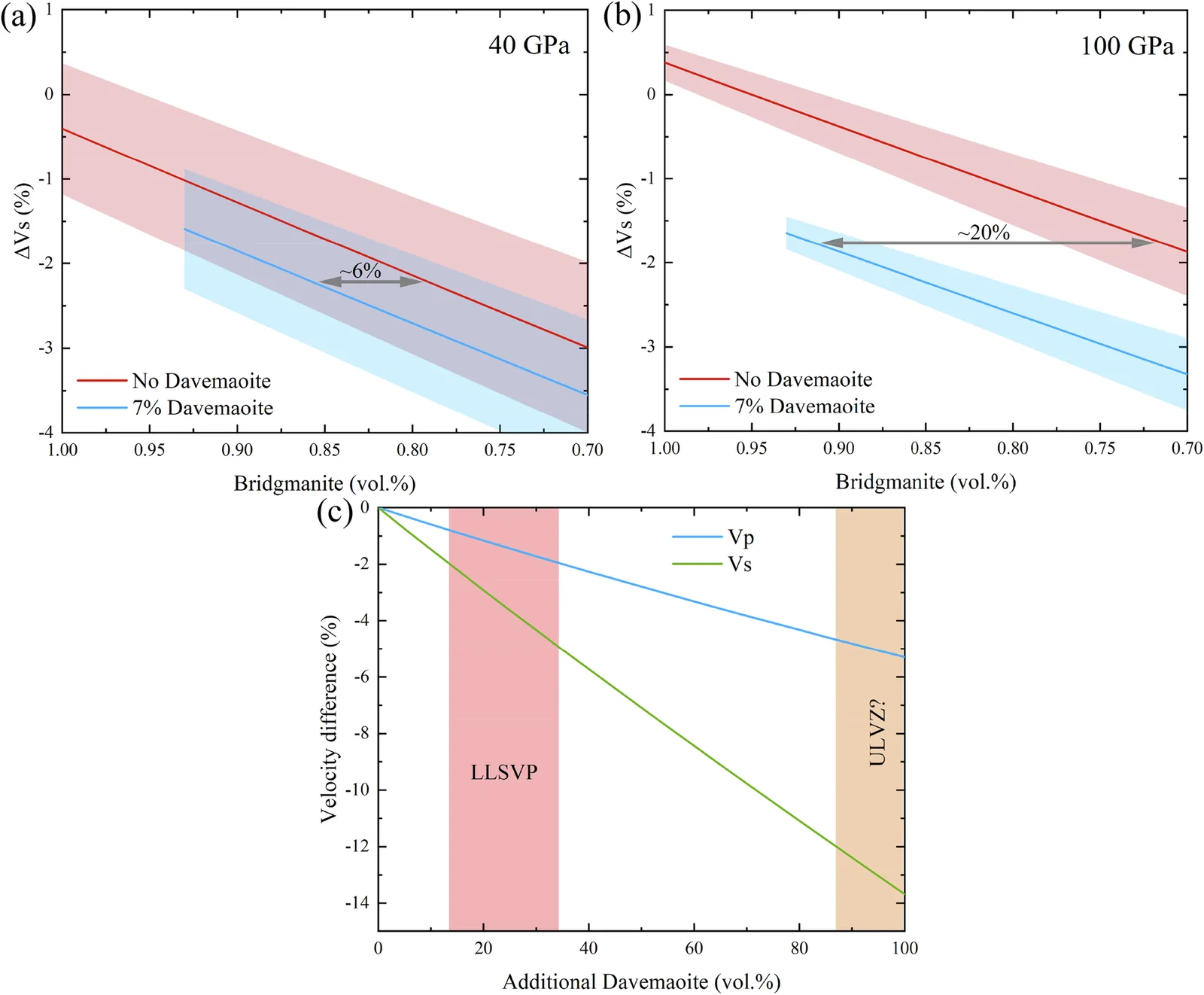
Effects of lower-mantle mineralogy on seismic velocities. **a**, **b** Shear-wave velocity deviations relative to the PREM model for varying bridgmanite abundances, with and without davemaoite, at 40 GPa (**a**) and 100 GPa (**b**) along an adiabatic mantle geotherm^27^. Shaded regions indicate standard deviations. **c** Predicted shear-wave velocity reductions resulting from enhanced davemaoite abundance near the base of the lower mantle.

To assess the influence of the updated elastic properties of cubic davemaoite on lower-mantle mineralogy, we calculated seismic velocity differences between mineralogical models with and without 7 vol.% davemaoite (Fig. 4). In Brillouin spectroscopy experiments using diamond anvil cells, which could measure sound velocity of minerals under lower mantle pressures, *Vp* of lower-mantle minerals often overlap with the *Vs* of diamonds^12,14,29^, making *Vs* the most robust observable. We therefore based our analysis exclusively on directly measured *Vs*.

Because reported elastic parameters for individual minerals vary substantially among studies, we adopted internally consistent fitting parameters for bridgmanite and ferropericlase derived from comprehensive datasets^13,15^ (Supplementary Table 3). Our objective is not to determine absolute mineral proportions, but rather to evaluate the relative impact of including or excluding davemaoite using the updated elastic properties determined in this study. These relative differences are less sensitive to uncertainties in the elastic properties of other phases. To avoid complications associated with the spin transition of ferropericlase in the mid-lower mantle^13,15^, we evaluated velocity variations as a function of bridgmanite abundance at 40 GPa, where ferropericlase is fully high-spin, and at 100 GPa, where it is fully low-spin (Fig. 4). The influence of the spin transition on the *G* of ferropericlase is relatively limited and does not produce abrupt changes or significant softening^15,30^. Therefore, treating ferropericlase as fully low-spin at 100 GPa should not introduce significant uncertainties into our model. The chemical composition of bridgmanite was fixed to Mg# = 93.5(11) with 4.5(12) wt.% Al_2_O_3_ at 40 GPa and Mg# = 95.0(5) with 3.3(3) wt.% Al_2_O_3_ at 100 GPa according to phase-equilibrium experiments conducted at similar pressures^2^. For ferropericlase, compositions of Mg# = 88.5(21) at 40 GPa and Mg# = 90.3(19) at 100 GPa were adopted. The variations in the elastic properties of bridgmanite and ferropericlase with Mg# and Al₂O₃ content are adopted from previous studies^13,15^, which were derived from fits to the currently available data.

As shown in Fig. 4a, b, along an adiabatic mantle geotherm^27^ the intrinsically low seismic velocities of davemaoite require a substantial increase in the abundance of the fast phase, bridgmanite, even when only a small fraction of davemaoite is present. Near the top of the lower mantle, reproducing the same shear-wave velocity as a davemaoite-free assemblage requires an increase in bridgmanite abundance of ~6 vol.%. This difference grows progressively with depth owing to the relatively small pressure dependence of the shear modulus of davemaoite. Near the base of the lower mantle, the bridgmanite proportion would need to increase by ~20 vol.% to offset the velocity reduction introduced by davemaoite. Given that most existing mineralogical models estimate bridgmanite abundances of ~75–90 vol.% in the lower mantle^14–16,29^, an additional ~20 vol.% increase is implausible. These results therefore imply that the normal lower mantle is likely depleted in davemaoite.

Fractional crystallization experiments of the Earth’s magma ocean indicate that the earliest crystallizing phases are Ca-bearing bridgmanite and ferropericlase^31^ leaving the residual melt progressively enriched in Ca. If such Ca-rich residual melts formed a basal magma ocean that subsequently crystallized to produce LLSVPs, which remain stable at the base of the lower mantle without participating in whole-mantle convection, the apparent depletion of davemaoite in the surrounding mantle could be compensated by its concentration within LLSVPs. For an extreme scenario in which the normal lower mantle contains almost no davemaoite, the CaSiO_3_ contents in the ambient mantle should lower than ~3%, according to recent experimental constraints on Ca solubility in bridgmanite^32^. Given that LLSVPs occupy ~3-8% of the mantle volume inferred from seismic observations^33,34^, an endmember scenario in which davemaoite is entirely absent from the ambient mantle would require its concentration within LLSVPs to be ~29-77% assuming a pyrolitic primitive mantle composition (~7% CaSiO_3_)^1^. For a chondritic primitive mantle composition (~5.5% CaSiO_3_)^13^, the corresponding upper boundary on davemaoite abundance in LLSVPs would be ~18–48%. As shown in Fig. 4c, davemaoite abundances of ~18–29% are sufficient to account for the observed 2–5% shear-wave velocity reductions characteristic of LLSVPs^19,20^. These results support a model in which davemaoite is heterogeneously distributed in the lower mantle, being depleted in the ambient mantle but strongly enriched within LLSVPs as a consequence of early magma-ocean differentiation.

Although enrichment in davemaoite may account for the observed properties of LLSVPs, alternative contributions, such as subducted slabs^6^ and thermal effects^35^, cannot be excluded. Given the complexity of Earth’s mantle, LLSVPs are likely influenced by multiple factors.

Enrichment of davemaoite within LLSVPs as a consequence of magma-ocean crystallization would impart a distinct geochemical signature to these deep-mantle reservoirs. During early crystallization, bridgmanite preferentially excludes incompatible elements (e.g., rare earth elements), leaving the residual basal magma ocean enriched in trace elements^36^. Relative to bridgmanite, davemaoite exhibits substantially higher partition coefficients for incompatible elements between crystal and melt, such that davemaoite crystallizing late from Ca-rich residual melts would become strongly enriched in these elements. In particular, the heat-producing elements K, U, and Th may become moderately to strongly compatible in davemaoite under deep lower-mantle conditions^36,37^. Concentration of these radiogenic elements within LLSVPs could generate localized internal heating, promoting thermal instabilities and facilitating plume initiation. Plumes rising from such chemically distinct reservoirs would carry characteristic geochemical signatures to the surface, potentially accounting for the enriched compositions observed in some ocean island basalts^38^.

## Methods

### Materials and sample

The starting glass sample is identical to the pure CaSiO₃ glass described in Zhou et al. ^6^. High-purity CaCO₃ powder (99.9%) was decarbonated by heating at 600, 700, 800, 900, and 1000 °C for 1 h at each temperature. The resulting CaO was then mixed with preheated SiO₂ powder (99.9%; 1000 °C overnight). The mixed powder was loaded into a platinum crucible, melted at 1600 °C for 30 min, and rapidly quenched to room temperature to produce a homogeneous glass. The chemical composition of the glass was analyzed using a JEOL JXA-8200 electron microprobe at the Institute of Meteoritics, University of New Mexico. The CaSiO_3_ contents are higher than 99.5%^6^. The glass samples were stored in a vacuum oven at 393 K until use.

High-temperature symmetric diamond anvil cells^39^ equipped with standard-cut diamond anvils were used to generate high pressures. Diamonds with 300 or 200 μm culets were mounted on Si₃N₄ or ZrO₂ seats. Sample chambers were prepared by drilling 100–120 μm diameter holes into pre-indented rhenium or tungsten gaskets with final thicknesses of 45–55 μm. The glass sample was loaded either sandwiched between NaCl layers or without any additional material. NaCl, or the glass itself, served as both the pressure-transmitting medium and thermal insulator. Pressure within the sample chamber was determined using the diamond anvil Raman edge^40^. After compression to the target pressure, samples were heated using a CO₂ laser for 10–30 min to synthesize davemaoite. Since all cells except C2 were used for only a single pressure condition, any texture development during compression has no influence on the results. Even for C2, which was measured at two pressure points, the sample was re-heated after further compression to resynthesize davemaoite.

Angle-dispersive X-ray diffraction (XRD) measurements were conducted at the PSICHE beamline of the SOLEIL Synchrotron using an incident X-ray wavelength of 0.3738 Å to characterize the synthesized phases. Supplementary Fig. 1 shows a representative XRD pattern collected at ~43 GPa and 300 K, in which all diffraction peaks can be indexed to davemaoite and NaCl. Although davemaoite is supposed to be tetragonal at room temperature^9,12^, due to the limited resolution, we did not see clear peak splitting.

### High-temperature Brillouin spectroscopy

Brillouin spectra were collected up to ~80 GPa and 973 K using a symmetric forward-scattering geometry with a scattering angle of ~50° and a 1.2 W, 532 nm single-mode diode-pumped solid-state laser. The scattering angle was calibrated to 50.1(1)° using a double-polished borosilicate crown optical glass (Supplementary Fig. 5)^41^. High temperatures were achieved by resistive heating in an Ar/H₂ atmosphere using a ceramic heater with nickel wires and were measured by R-type thermocouples attached to the diamond anvils, with a typical uncertainty of 1–2%^39^. When the Brillouin laser was focused on the sample chamber without external heating, the thermocouple readings increased by 1–2 °C, indicating that the thermocouples were sufficiently sensitive to temperature variations within the sample chamber. Because the sample is transparent at the Brillouin laser wavelength, laser-induced heating is expected to be minimal. Nevertheless, future investigations of the laser-power dependence of sound velocity would help to further assess and quantify any potential heating effects. The double stage seats^39^ allow that the heater thickness (~5 mm) exceeds the combined thickness of the two diamond anvils (~4 mm), ensuring a homogeneous temperature distribution across the sample chamber. Pressures at high temperatures were determined in situ using the diamond anvil Raman edge, following previously established calibrations^40,42^. Details of the pressure uncertainty estimation are provided in Supplementary Note 1.

At room temperature, Brillouin spectra were collected at 5–6 different χ angles at 30° intervals. The resulting velocity variations are within 60 m/s, comparable to the typical uncertainties of single-crystal Brillouin and ultrasonic measurements^4,15^ (Supplementary Fig. 2). Given the pronounced elastic anisotropy of tetragonal davemaoite^5,7^, such small variations indicate a negligible effect of CPO, consistent with the XRD patterns shown in Supplementary Fig. 1. For high-temperature measurements, only a single χ angle was measured due to the difficulty of rotating the diamond anvil cell. Since theoretical studies consistently show that room-temperature tetragonal davemaoite is more elastically anisotropic than cubic davemaoite^5,7^, the negligible CPO observed in the tetragonal phase suggests that CPO effects should also have minimal influence on the high-temperature measurements. Because diamond anvils are prone to failure during prolonged measurements at the highest temperatures, each cell was used for a single pressure condition, except for the lowest-pressure run, which was used for two pressure points. Prior to all measurements, the entire cell assembly was annealed at 250 °C for 48 h to reduce differential stress. Representative Brillouin spectra acquired with integration times of 10–30 min at different temperatures are shown in Supplementary Fig. 3. The sharpness of the Brillouin peaks and the consistency of room-temperature velocities with previous studies^3,12^ (Fig. 1) indicate that differential stress effects are negligible.

To assess the reproducibility of our results, in addition to testing different seat materials, we performed high-temperature measurements at ~37 GPa (C6P1 in Supplementary Table 1) during both heating and cooling cycles. The consistent behavior observed between heating and cooling, as well as across different seat materials, demonstrates the high reproducibility of our measurements.

### High-pressure fittings

By combining volume data from previous laser-heating X-ray diffraction measurements (Supplementary data 1)^3,4,10,22–24^ with the *Vs* data obtained in this study, the elastic properties of cubic davemaoite were determined. The third-order Birch-Murnaghan EOS^25^ and the third-order Eulerian finite-strain EOS^26^ were applied for volume and velocity, respectively, under the Mie-Grüneisen-Debye framework^26^:

1 $$P\left(V,T\right)=\frac{3}{2}K_{t0}\left[{\left(\frac{{V}_{0}}{V}\right)}^{\frac{7}{3}}-{\left(\frac{{V}_{0}}{V}\right)}^{\frac{5}{3}}\right]\left[1+\frac{3}{4}({K}_{t0}^{{\prime} }-4) \left({\left(\frac{{V}_{0}}{V}\right)}^{\frac{2}{3}}-1\right)\right]+\gamma \Delta {U}_{q}/V$$

2 $${K}_{S}={K}_{t}(1+\alpha \gamma T)$$

3 $${K}_{S}\left(V,T\right)=	 {(1+2f)}^{\frac{5}{2}}[{K}_{S0}+\left(3{K}_{S0}{K}_{S0}^{{\prime} }-5{K}_{S0}\right)f+13.5({K}_{S0}{K}_{S0}^{{\prime} }-4{K}_{S0}){f}^{2}] \\ 	+\left(\gamma+1-{q}_{0}\right)\gamma \Delta {U}_{q}/V-{\gamma }^{2}\Delta ({C}_{V}T)/V$$

4 $$G\left(V,T\right)=	 {(1+2f)}^{\frac{5}{2}}[{G}_{0}+\left(3{K}_{S0}{G}_{0}^{{\prime} }-5{G}_{0}\right)f+(6{K}_{S0}{G}_{0}^{{\prime} }-24{K}_{S0}-14{G}_{0} \\ 	+4.5{K}_{S0}{K}_{S0}^{{\prime} }){f}^{2}]-{\eta }_{s}\Delta {U}_{q}/V$$

5 $$f=0.5\left[{\left(\frac{{V}_{0}}{V}\right)}^{\frac{2}{3}}-1\right]$$

where *Ks* is the adiabatic bulk modulus, *Kt* the isothermal bulk modulus, *G* the shear modulus, *V* the volume, and *T* the temperature. The parameters *α, γ, q*_*0*_, and *C*_*V*_ denote the thermal expansion coefficient, Grüneisen parameter, its volume derivative, and the heat capacity, respectively. *η*_*s*_ is the shear strain derivative of γ, and *Uq* is the internal energy. The symbol *Δ* represents the difference between high-temperature conditions and the reference temperature (300 K for this study). The subscript “0” indicates the values at ambient conditions. *γ* and *α* can be calculated using equations: *γ* = *γ*_*0*_ *(V/V*_*0*_*)*^*q0*^ and $\alpha=1/V{(\partial V/\partial T)}_{P}$. The *Uq*, *C*_*V*_, and *η*_*s*_ are calculated via:

6 $${U}_{q}\left(V,T\right)=9{nRT}{\left(\frac{{\theta }_{D}}{T}\right)}^{-3}{\int }_{0}^{\frac{{\theta }_{D}}{T}}\frac{{x}^{3}}{{e}^{x}-1}{dx}$$

7 $${\theta }_{D}={\theta }_{0}{e}^{-\frac{\gamma -{\gamma }_{0}}{{q}_{0}}}$$

8 $${\eta }_{s}={\eta }_{s0}\frac{V}{{V}_{0}}$$

9 $${C}_{V}\left(V,T\right)=9{nR}{\left(\frac{{\theta }_{D}}{T}\right)}^{-3}\int _{0}^{\frac{{\theta }_{D}}{T}}\frac{{x}^{4}{e}^{x}}{{({e}^{x}-1)}^{2}}{dx}$$

where *n* is the number of atoms in the chemical formula, *R* the gas constant, and *θ*_*D*_ the Debye temperature.

### Seismic properties of multi-phase assemblies

The density and elastic moduli of a multi-phase assembly are calculated using Hill average^43^:

10 $$\rho=\sum _{i}{f}_{i}\rho _{i}$$

11 $${M}^{V}=\sum _{i}{f}_{i}M_{i}$$

12 $${M}^{R}={\left(\sum _i{f}_{i}{M}_{i}^{-1}\right)}^{-1}$$

13 $${M}^{{VRH}}=0.5({M}^{R}+{M}^{V})$$

where *ρ*_*i*_, *M*_*i*_, and *f*_*i*_ are the density, the elastic moduli (*K* or *G*), and the volume fraction of component i, respectively. The superscript V, R, and VRH are the upper bound (isostrain), lower bound (isostress), and Hill average, respectively. Then the seismic velocities could be calculated using the density and elastic moduli.

## Supplementary information

Supplementary Information

Description of Additional Supplementary Files

Supplementary Data 1

Transparent Peer Review file

## Data Availability

The data generated in this study are provided in the Supplementary Information.
