# Supplementary material for: Depletion of davemaoite in Earth’s ambient lower mantle
Source: Nat Commun. 2026 Aug 15;17:9817. doi: 10.1038/s41467-026-76943-0 (PMC13575100; doi:10.1038/s41467-026-76943-0)
Supplement: Supplementary file 1 [file 41467_2026_76943_MOESM1_ESM.pdf]

Supplementary Information for

Davemaoite Depletion in the Ambient Lower Mantle

Ming Hao[1*], Motohiko Murakami[1], Pinku Saha[1], Guignot Nicolas[2]

[1]Department of Earth and Planetary Sciences, Institute of Geochemistry and Petrology, ETH Zürich, Zürich, Switzerland

[2]Soleil Synchrotron, French National Centre for Scientific Research, France

*Corresponding author: Ming Hao (ming.hao@eaps.ethz.ch)

This Supplementary Information includes:

Supplementary Notes 1 to 3

Supplementary Figures 1 to 6

Supplementary Tables 1 to 4

Supplementary Note 1. Pressure uncertainty estimation

The focus and positioning of the laser can influence pressure measurements. In this study, we performed in-situ Raman measurements using the laser spot as the Brillouin. Therefore, when the Brillouin signal is well focused on the sample, the Raman measurements are also well aligned. As a result, the measured pressure directly corresponds to the location from which the Brillouin spectra are collected, minimizing uncertainties associated with pressure gradients within the sample chamber. In addition, it is unnecessary to apply an additional correction of thermal pressure for externally resistive heating. During high-temperature experiments, the entire diamond anvils are heated, and pressures are measured directly from the hot anvils using calibrations established under comparable conditions[1]. As such, thermal effects are inherently accounted for in the measured pressures.

The uncertainties in pressure primarily arise from the fitting of the diamond Raman edge and the coupled effects of temperature. The Raman edge position is temperature dependent, and temperature uncertainties measured by thermocouples are typically on the order of 10–20 K for temperatures up to ~1000 K. These combined effects result in an estimated pressure uncertainty of ~0.2–0.3 GPa at ~900 K. In addition, the limited spectral resolution of our in-situ Raman system (~1 cm^{-1}) introduces uncertainties of ~0.4–0.5 GPa. Consequently, as summarized in Table S1, the overall pressure uncertainty for most high-temperature measurements is ~0.5 GPa. For experiments conducted at higher pressures and temperatures, non-ideal diamond conditions can further increase the fitting uncertainty to ~1–2 GPa. In contrast, room-temperature pressures were measured using a higher-resolution Raman system (~0.3 cm^{-1}) with a smaller focal spot (3–4 μm), resulting in reduced fitting uncertainties of ~0.2–0.5 GPa.

To evaluate the influence of pressure uncertainties on the fitting results for cubic davemaoite, we performed an additional test in which all pressures were reduced by one standard deviation prior to refitting. This represents an extreme scenario, as the uncertainties would be expected to be more evenly distributed rather than systematically biased toward lower values. The fitting gives K_{t0} = 243(4) GPa, K_{t0}' = 4.09(8), G_0 = 118(2) GPa, G_0' = 1.45(2), θ_0 = 1450(300) K, γ_0 = 2.0(2), q_0 = 1.1(4), and η_0 = 2.5(13). Compared with the values reported in the main text, only G_0' is marginally larger (1.45 VS 1.44) and η_0 slightly smaller (2.5 VS 2.8), both well within their respective uncertainties. These results indicate that the effects of pressure uncertainties are adequately captured within the fitting uncertainties and have a negligible impact on the overall conclusions.

Supplementary Note 2. Velocity uncertainty estimation

Fig. S3 shows the raw Brillouin spectra without any smoothing or filtering. For the sharp peaks observed at lower temperatures, the fitting uncertainty is typically ~0.02–0.03 GHz, corresponding to ~10–20 m/s in sound velocity. At higher temperatures (e.g., 973 K), the broader signals yield fitting uncertainties of ~0.05–0.06 GHz, corresponding to ~30–40 m/s. Because all high-temperature measurements were collected at the same sample position and geometry, neither

angle-calibration uncertainties nor velocity variations with χ angle are expected to influence the observed temperature-dependent velocity trends. Therefore, the velocity uncertainties reported in the table primarily reflect the fitting uncertainties of the Brillouin peaks and the small differences between the Stokes and anti-Stokes signals. Even considering an angle-calibration uncertainty of 0.1°, the resulting velocity uncertainty would be only ~10 m/s and would not significantly affect the conclusions.

Supplementary Note 3. Velocity corrections

The velocity corrections shown in Fig. 1b for 300 K tetragonal davemaoite and high-temperature cubic davemaoite are based on the fitted pressure dependences obtained in this study. Because the pressure dependences of the tetragonal and cubic phases differ slightly, the velocity corrections for temperatures above 300 K but below the phase boundary were estimated by assuming a linear variation in pressure dependence with temperature.

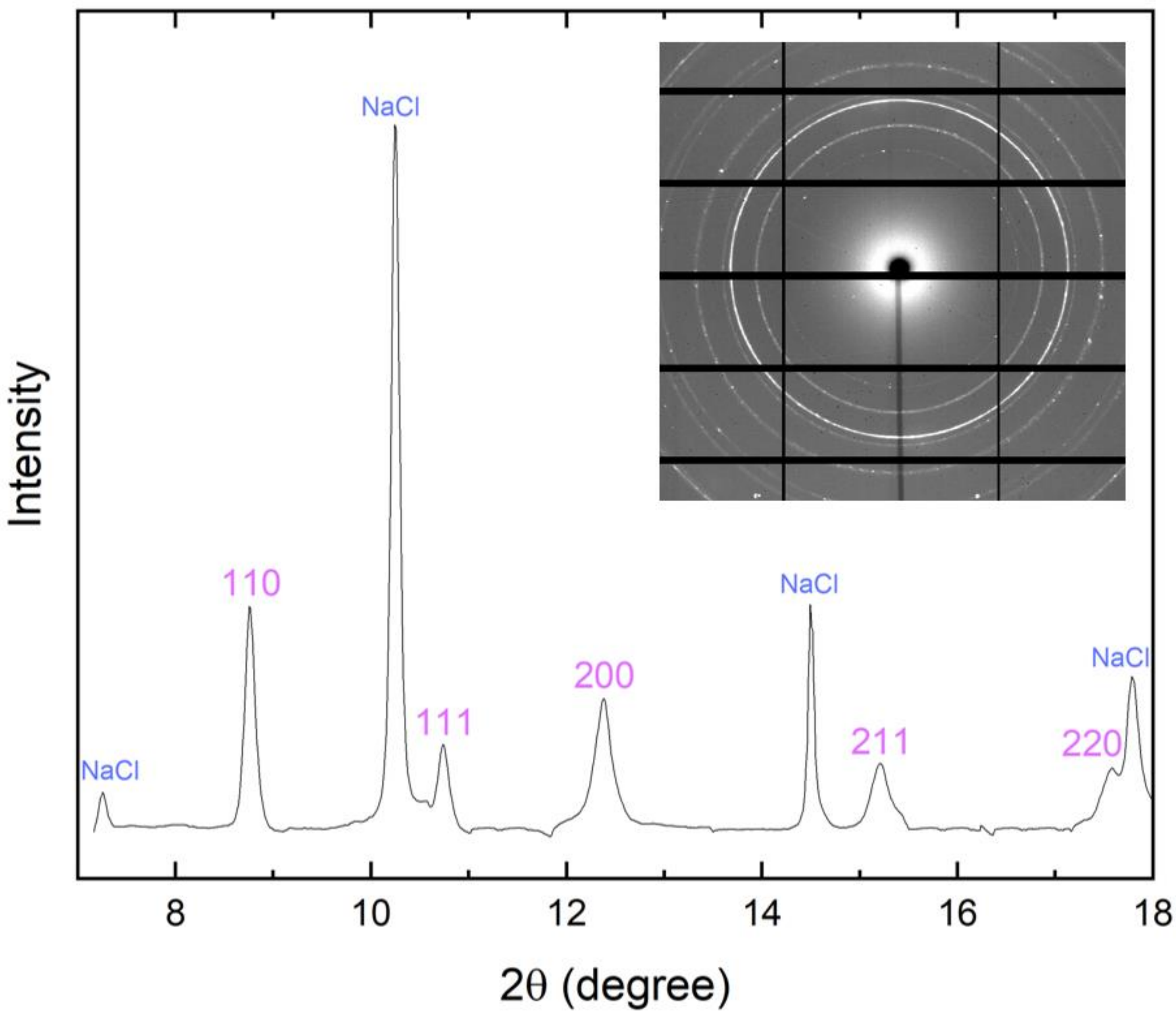

Supplementary Figure 1. XRD spectra of synthesized davemaoite at ~43 GPa and 300 K. All the peaks could be labeled by davemaoite and NaCl, but the limited resolution prohibits further structure refinement of davemaoite. The raw pattern indicates negligible crystal preferred orientations.

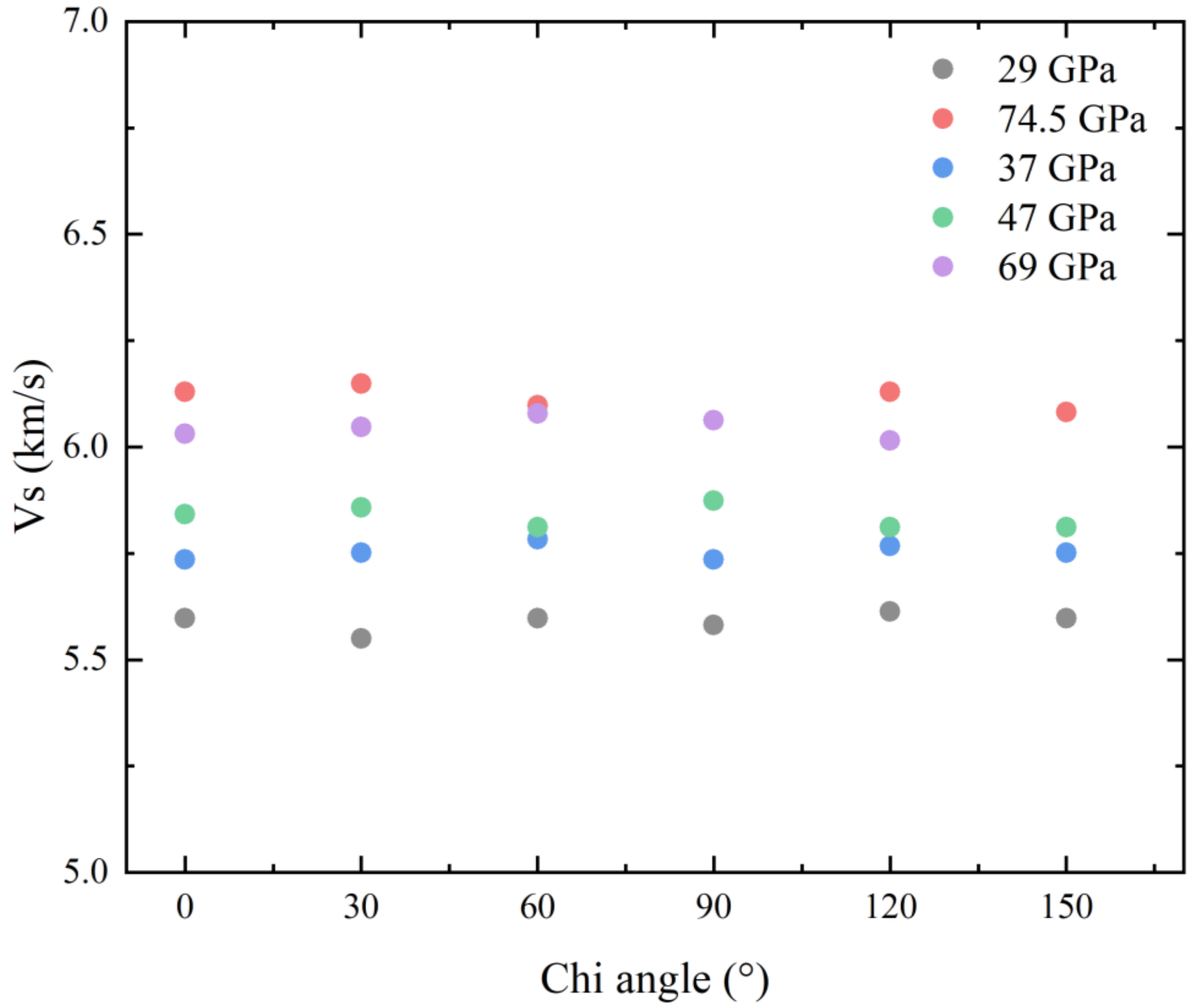

Supplementary Figure 2. The variation of shear wave velocities at different Chi angles at different pressures.

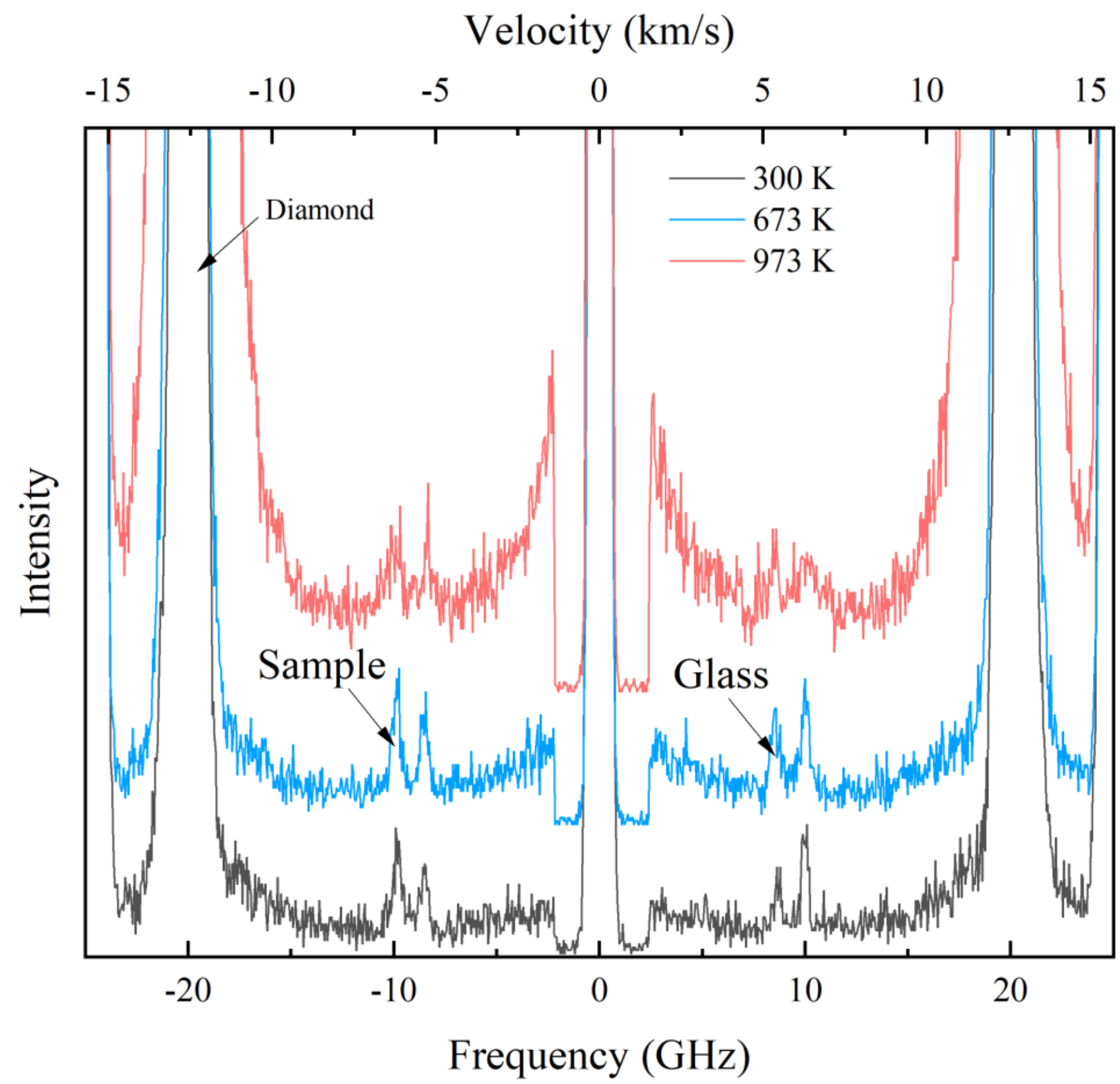

Supplementary Figure 3. Typical Brillouin spectra of the sample at ~80 GPa and different temperatures. Only the Vs of sample was observed, and the Vp of sample overlaps with the Vs of diamond.

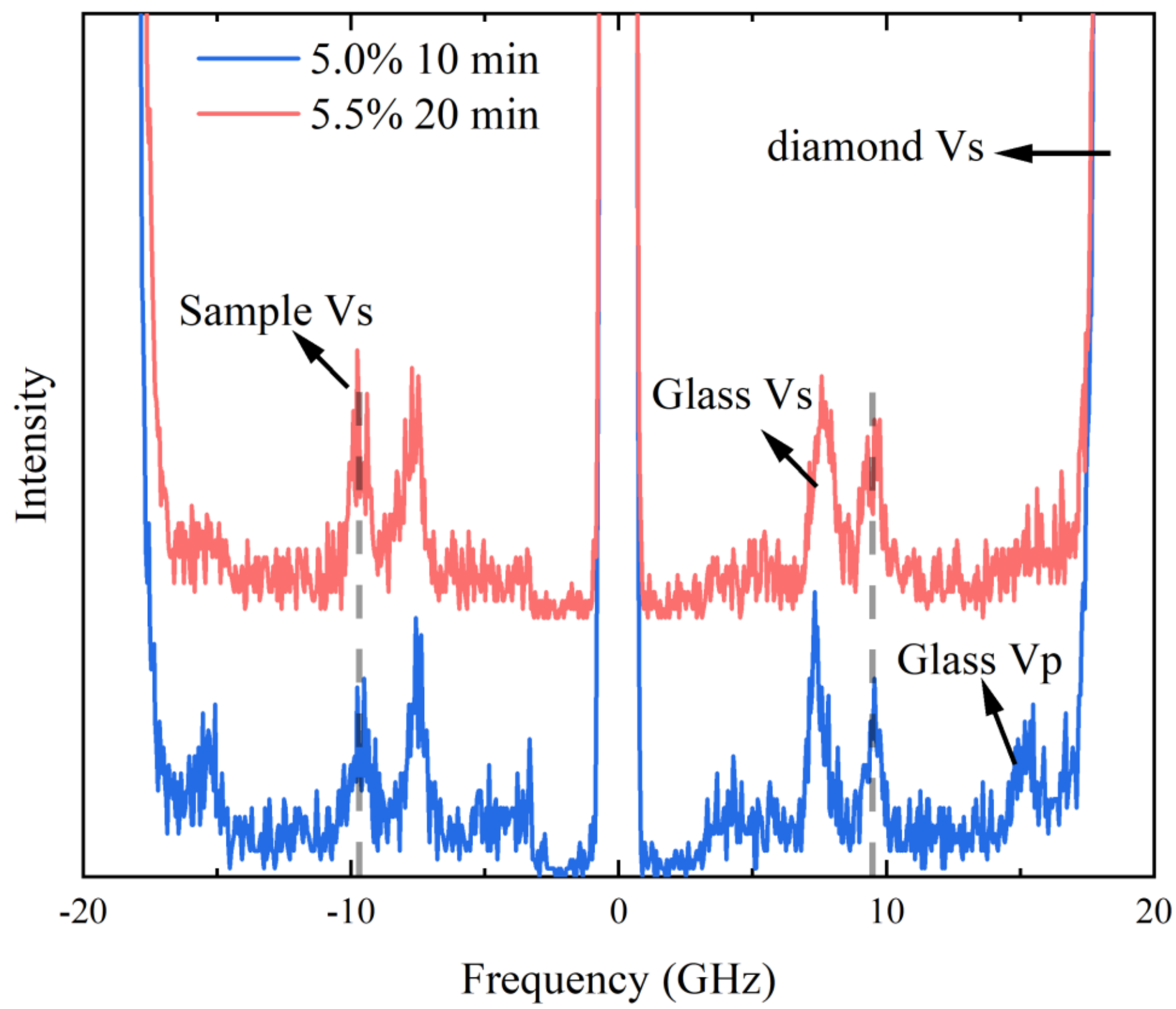

Supplementary Figure 4. Comparison of Brillouin spectra collected after heating under different laser powers and heating durations. The values of 5% and 5.5% represent the percentage of the total CO_2 laser power used during heating. Despite variations in heating time and laser power, the sample peaks remain consistent, indicating that the presence of residual glass has no influence on the measured sound velocities.

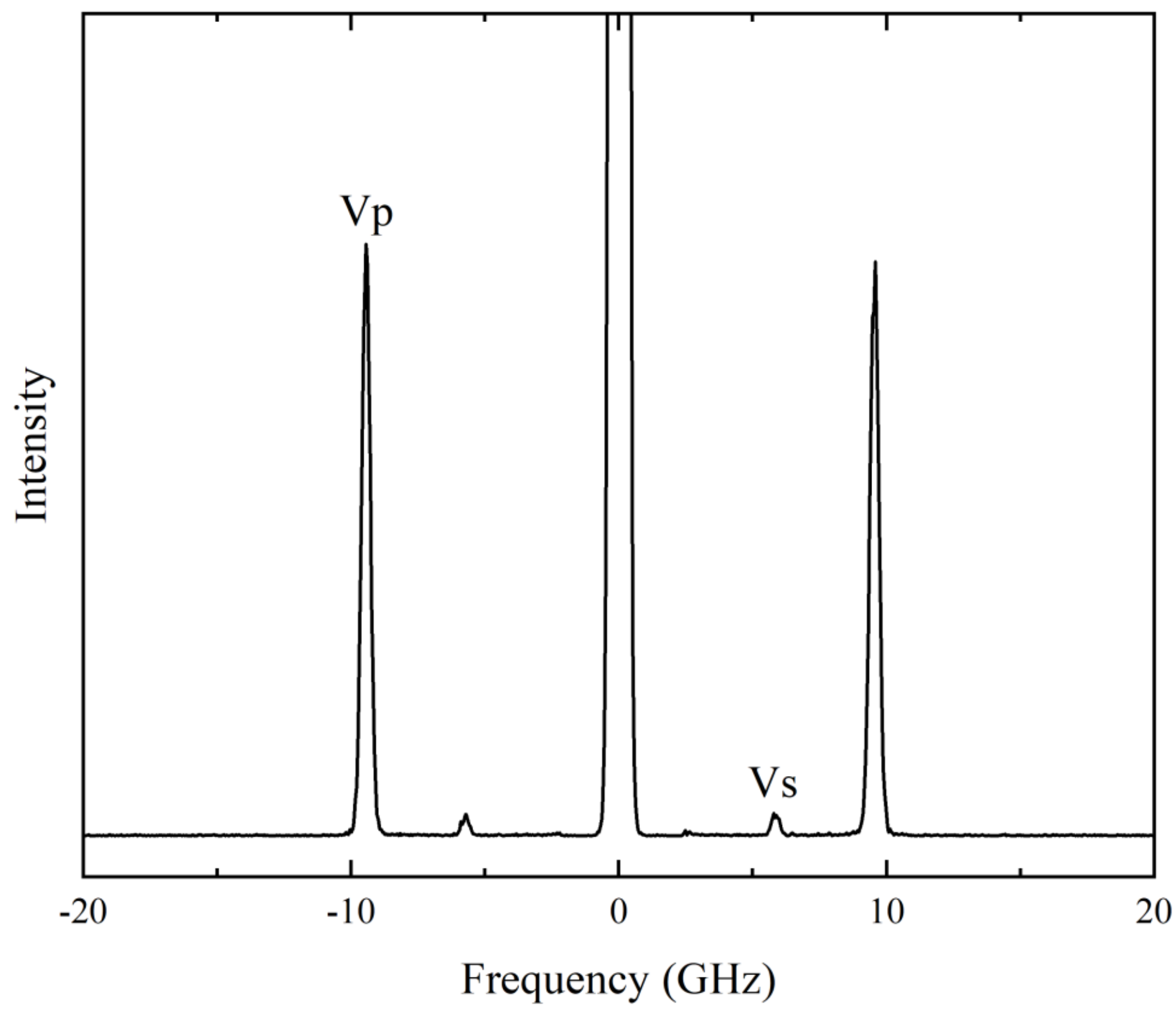

Supplementary Figure 5. Representative Brillouin spectra of borosilicate crown optical glass (BK7) used for scattering-angle calibration. A 50 µm-thick, double-side-polished BK7 glass wafer with a well-characterized velocity (Vp = 5965 m/s) determined from high-precision ultrasonic measurements was placed at the sample position and measured under the same experimental geometry. The resulting Brillouin frequency shifts were used to calibrate the actual scattering angle[2]. The uncertainty in the calibrated angle was determined from the uncertainty in fitting the Brillouin peaks of the BK7 standard.

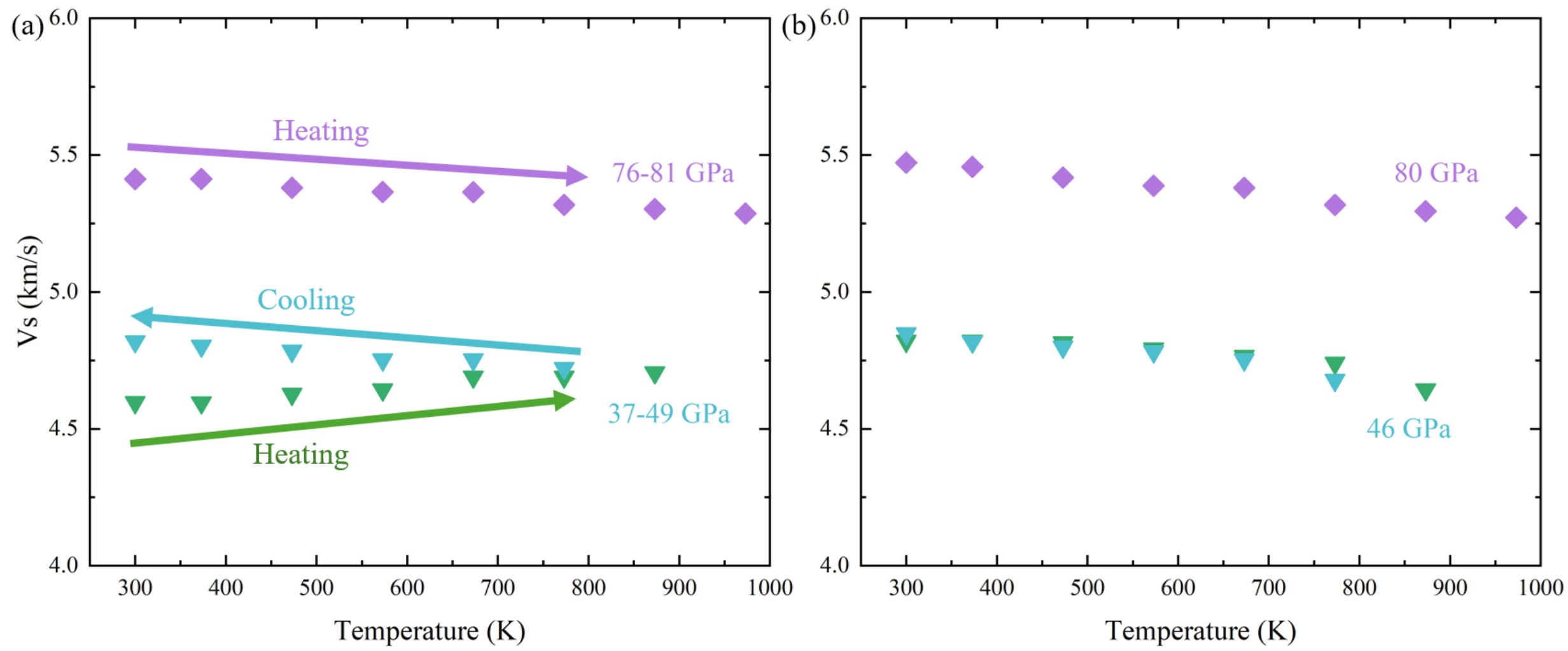

Supplementary Figure 6. Representative Vs measurements of the glass collected together with the sample under high-temperature conditions. (a) Measured Vs of the glass during heating and cooling, without correction for pressure variations induced by temperature changes. (b) Vs corrected using the room-temperature pressure dependence from previous experiments[3]. The monotonic variation of the glass velocities demonstrates the reliability of the measurements and confirms that the anomalous velocity behavior observed in davemaoite is intrinsic and associated with the phase transition rather than an experimental artifact.

Run #	T (K)	P (GPa)	Uncertainty (GPa)	Vs (m/s)	Uncertainty (m/s)
C1P1	300	74.5	0.5	6120	50
C2P1	300	29	0.2	5591	20
C2P1	373	29.5	0.5	5610	20
C2P1	473	28.5	0.5	5626	30
C2P1	573	28.5	0.5	5689	30
C2P1	673	27.5	0.5	5686	20
C2P1	729	27.5	0.5	5654	20
C2P1	773	26	0.5	5623	50
C2P1	873	27.5	0.5	5623	30
C2P1	300	30	0.2	5626	30
C2P2	300	47	0.3	5843	20
C2P2	373	46.5	0.5	5855	50
C2P2	473	45.5	0.5	5887	30
C2P2	523	45	0.5	5902	30
C2P2	573	43	0.5	5902	30
C2P2	623	43.5	0.5	5934	30
C2P2	673	42.5	0.5	5918	20
C2P2	723	42.5	0.5	5902	30
C2P3	300	57.5	0.5	5968	20
C3P1	300	49	0.3	5827	30
C3P1	373	50	0.5	5871	50
C3P1	473	51	0.5	5918	50
C3P1	573	52	0.5	5996	40
C3P1	673	55	1.0	6028	60
C3P1	773	58.5	1.0	6044	60
C4P1	300	69	0.5	6028	30
C4P1	373	69.5	1.0	6075	20
C4P1	473	68	1.0	6122	30
C4P1	573	66.5	1.0	6138	50
C4P1	673	65	1.0	6154	40
C4P1	773	64	1.0	6138	50
C4P1	873	64	1.5	6122	60
C5P1	300	76	0.5	6185	30
C5P1	373	77	1.0	6216	30
C5P1	473	77.5	1.0	6251	30
C5P1	573	78.5	1.0	6282	30
C5P1	673	79	1.0	6301	20
C5P1	773	80	1.0	6285	30
C5P1	873	80.5	1.5	6270	40
C5P1	973	81	2.0	6251	50
C5P1	300	63.5	2.0	5996	50
C6P1	300	37	0.3	5736	20
C6P1	373	37	0.5	5767	30

C6P1	473	38.5	0.5	5830	30
C6P1	573	40	0.5	5883	30
C6P1	673	43	0.5	5915	30
C6P1	773	44	0.5	5899	50
C6P1	873	48.5	0.5	5931	50
C6P1	773	47.5	0.5	5931	50
C6P1	673	46	0.5	5946	40
C6P1	573	45	0.5	5931	40
C6P1	473	45.5	0.5	5915	30
C6P1	373	45.5	0.5	5849	30
C6P1	300	45	0.4	5830	30

Supplementary Table 1. The measured Vs of davemaoite in this study. The typical uncertainty for temperature is 1-2%.

Reference	V_0(Å^3)	Kt_0 (GPa)	Kt_0'	G_0(GPa)	G_0'	θ_0(K)	γ_0	q_0	η_{s0}
This study	45.58	243(4)	4.08(8)	118(2)	1.44(2)	1450(300)	2.0(2)	1.1(4)	2.8(13)
Gréaux et al.[4]	45.4	248	4	126	1.6	1000	1.42	2.65	1.54
Thomson et al.[5]	45.57(2)	248(3)	3.6(1)	107(1)	1.66(22)	771(90)	1.67(4)	1.1(2)	3.3

Supplementary Table 2. Thermoelastic parameters of cubic davemaoite (pure $CaSiO_3$) from different experimental studies. The value for Gréaux et al.[4] are from the refitting in Fu et al.[6] using the same EOS as this study. Previous studies did not distinguish the small difference between *Kt* and *Ks*, which should not result in obvious difference.

Mineral	V_0(Å^3)	Kt_0 (GPa)	Kt_0'	$G_0(GPa)$	G_0'	$\theta_0(K)$	γ_0	q_0	η_{s0}
Ferropericlase[6,7]	-	-	4	-	-	686	1.39	1.7	2.2
Davemaoite	45.58	243	4.09	118	1.44	1450	2.0	1.1	2.8
Bridgmanite[6,7]	-	-	-	-	-	905	1.57	1.1	2.6

Supplementary Table 3. Thermoelastic parameters of lower mantle mineral phases. The compositional dependent volume and bulk modulus of ferropericlase and davemaoite are from the fitting in Fu et al.[6] and shear modulus is from the fitting in Murakami et al.[7].

Reference	V_0(Å^3)	Ks (GPa)	Ks_0'	G_0(GPa)	G_0'
This study	45.58	228(5)	3.7(4)	116(3)	1.23(2)
Gréaux et al.[4]	45.58	228(5)	3.7(4)	116(3)	1.3(2)
Kudo et al.[8]	45.58	248	4.0	116(2)	1.20(2)

Supplementary Table 4. Elastic parameters of tetragonal davemaoite (pure $CaSiO_3$) from different experimental studies. For consistence, we used the same equations as those employed by Gréaux et al.[4] and Kudo et al[8], which differ slightly from the equations used to fit the high-temperature data described in the Methods section. Due to the lack of data, the ambient-pressure properties and bulk modulus in this study were directly adopted from Gréaux et al.[4].

Supplementary References

1. Ono, S., Mibe, K. & Ohishi, Y. Raman spectra of culet face of diamond anvils and application as optical pressure sensor to high temperatures. *Journal of Applied Physics* **116**, 053517 (2014).
2. Murakami, M., Asahara, Y., Ohishi, Y., Hirao, N. & Hirose, K. Development of *in situ* Brillouin spectroscopy at high pressure and high temperature with synchrotron radiation and infrared laser heating system: Application to the Earth's deep interior. *Physics of the Earth and Planetary Interiors* **174**, 282–291 (2009).
3. Geballe, Z. M., Arveson, S. M., Speziale, S. & Jeanloz, R. Sound speed and refractive index of amorphous CaSiO3 upon pressure cycling to 40 GPa. *American Mineralogist* **107**, 2212–2218 (2022).
4. Gréaux, S. *et al.* Sound velocity of CaSiO3 perovskite suggests the presence of basaltic crust in the Earth's lower mantle. *Nature* **565**, 218–221 (2019).
5. Thomson, A. R. *et al.* Seismic velocities of CaSiO3 perovskite can explain LLSVPs in Earth's lower mantle. *Nature* **572**, 643–647 (2019).
6. Fu, S., Zhang, Y., Okuchi, T. & Lin, J.-F. Single-crystal elasticity of (Al,Fe)-bearing bridgmanite up to 82 GPa. *American Mineralogist* **108**, 719–730 (2023).
7. Murakami, M., Khan, A., Sossi, P. A., Ballmer, M. D. & Saha, P. The Composition of Earth's Lower Mantle. *Annual Review of Earth and Planetary Sciences* **52**, 605–638 (2024).
8. Kudo, Y. *et al.* Sound velocity measurements of CaSiO3 perovskite to 133 GPa and implications for lowermost mantle seismic anomalies. *Earth and Planetary Science Letters* **349–350**, 1–7 (2012).
