# Supplementary material for: Depletion of davemaoite in Earth’s ambient lower mantle
Source: Nat Commun. 2026 Aug 15;17:9817. doi: 10.1038/s41467-026-76943-0 (PMC13575100; doi:10.1038/s41467-026-76943-0)
Supplement: Supplementary file 2 [file 41467_2026_76943_MOESM2_ESM.pdf]

Description of Additional supplementary files

Supplementary Data 1: All the XRD measurements used to fit the thermoelastic properties of cubic davemaoite.
