# Supplementary material for: Depletion of davemaoite in Earth’s ambient lower mantle
Source: Nat Commun. 2026 Aug 15;17:9817. doi: 10.1038/s41467-026-76943-0 (PMC13575100; doi:10.1038/s41467-026-76943-0)
Supplement: Supplementary file 3 [file 41467_2026_76943_MOESM4_ESM.pdf]

nature portfolio

Peer Review File

Depletion of Davemaoite in Earth’s Ambient Lower Mantle

Corresponding Author: Dr Ming Hao

This file contains all reviewer reports in order by version, followed by all author rebuttals in order by version.

Version 0:

Reviewer comments:

Reviewer #1

(Remarks to the Author)
Hao et al measure the elastic properties and P-T slope second-order cubic-tetragonal transition in $CaSiO_3$ perovskite (Davemaoite) to 80 GPa and 973 K using Brillouin spectroscopy in a resistively heated diamond anvil cell. Their estimates of the P-T slope and position of the transition are consistent with lower pressure (including larger volume) measurements.

Uncertainties in the cubic-tetragonal transition in $CaSiO_3$ have plagued deep Earth geophysics for decades, and this study more or less puts the question to rest: At plausible lower mantle P-T conditions, only the cubic phase appears to stable. Of course there are always some lingering caveats. For example, DACS are plagued by deviatoric stresses, may be sensitive to deviatoric stress owing to its expression as a lattice distortion. However, I think the present experimental approach is the best one can hope for to mitigate most issues.

Less certain is the extrapolation of elastic properties to higher temperatures (well above the experimental conditions), however this is a longstanding issue that relates to technical limitations of the Brillouin spectroscopy approach. Nevertheless, the authors have admitted to generous uncertainties and I do not find their estimates to be over-stated beyond the support of the underlying data. In terms of known-unknowns, I would consider the spin transition in ferropericlase to be a significant source of uncertainty, particularly its potential influence upon Fe partitioning.

The interpretation of LLSVP as cubic Ca-Pv (davemaoite)-rich following fractionation of a basal magma ocean is plausible, as it is last to crystallize from any plausible starting composition. The depletion of the remainder of the lower mantle in CaO is also possible, although it also requires some underlying rationale such as containing cumulates of the magma ocean and/or the BEAMS hypothesis of Ballmer et al. (2017). In any case I think the lower mantle chemical composition is poorly constrained and I welcome ideas such as those presented in this paper.

There is only one minor point that I would raise, which is the claim that LLSVP comprise ~8 vol% of the mantle. In fact, the cited study does not aim to measure LLSVP alone, but rather a broader class of lower velocity anomalies. Instead, the vast majority of studies aiming to constrain LLSVP volume specifically (my recent student catalogued these to compare with his own estimate) find it to be only 2-3% of the total mantle (similar to continents). The LLSVP appear large in map view, however, it is important to note that the CMB is ~4x smaller in area than Earth's surface (accordingly, LLSVP are estimated to be roughly ~3-4x thicker than continents, extending ~600-800 km above the CMB at their peaks).

Sincerely,
John Hernlund
(Nozomi 163, 15 March 2026)

Reviewer #2

(Remarks to the Author)
This manuscript presents new high-pressure and high-temperature Brillouin spectroscopy measurements on $CaSiO_3$ davemaoite, providing important constraints on the tetragonal-cubic phase transition and its elastic properties under lower mantle conditions. By combining these new data with previous experimental results, the authors refine the phase boundary and derive updated thermoelastic parameters, which have significant implications for interpreting seismic observations and understanding lower mantle mineralogy and heterogeneity. In particular, the conclusion that tetragonal davemaoite is unlikely to be stable along any plausible mantle geotherm, and the suggestion that davemaoite may be depleted in the

ambient lower mantle but enriched in LLSVPs, are both scientifically meaningful and relevant to current debates in deep Earth geophysics.
Overall, the study is of clear scientific value and suitable for publication. However, there are several points that require clarification or further discussion. Addressing these issues would improve the rigor and clarity of the manuscript. I therefore recommend acceptance after revision.
Specific comments
1. The tetragonal and cubic perovskite structures of $CaSiO_3$ are very similar, and their XRD patterns differ only subtly. In Extended Data Fig. 1, the XRD pattern was collected at ~43 GPa, but the temperature is not specified. I assume that this measurement was performed at room temperature. If so, $CaSiO_3$ should adopt the tetragonal perovskite structure under these conditions. However, all diffraction peaks in the figure are indexed as belonging to the cubic structure, which may not be appropriate. The authors are encouraged to clarify the experimental temperature and justify the structural assignment.
2. The authors determine the phase transition based on the temperature dependence of Vs shown in Fig. 1(b), which is consistent with approaches used in previous studies. However, I wonder whether the authors have collected XRD patterns for the sample both before and after the phase transition. Is it possible to directly identify the phase transition from XRD data? Although the structural differences between tetragonal and cubic $CaSiO_3$ are subtle, the tetragonal phase may exhibit peak splitting and weak reflections compared to the cubic phase. Any additional structural evidence from XRD would strengthen the conclusions.
3. The pressure in the DAC experiments was determined using the Raman edge of diamond anvils. This method may introduce relatively large uncertainties at low pressures, depending on the laser focus position within the sample chamber and its depth relative to the diamond culet surface. In addition, during heating at high pressure, thermal pressure can develop, causing the pressure to increase with temperature. Although the authors mention that pressure corrections were applied during heating, it is not entirely clear how uncertainties arising from (i) Raman pressure calibration and (ii) thermal pressure effects were treated. Were these uncertainties explicitly incorporated into the further high-pressure fitting (third-order Birch-Murnaghan and Eulerian finite-strain EoS)? If so, how significant is their impact on the fitted parameters and final conclusions? A more detailed explanation would be helpful.
4. In Fig. 2(b), the phase boundary labeled as “Sun et al. (2016)” may be misleading. That study did not explicitly define a phase boundary, but rather showed that $CaSiO_3$ is cubic above ~1200 K. Therefore, including this line could cause confusion, and I suggest removing it.
5. In Fig. 3(b), the authors present Vp and Vs of cubic $CaSiO_3$ at 1500 K and 2500 K, along with extrapolated results from previous low-pressure studies. There are noticeable discrepancies between the present results and previous extrapolations for both Vp and Vs. What is the origin of these differences? The manuscript does not seem to provide sufficient discussion on this point. It would be useful for the authors to elaborate on the reasons for these discrepancies, such as differences in pressure range, fitting strategy, or thermoelastic parameters.

Reviewer #3

(Remarks to the Author)
Hao and co-authors present a manuscript that primarily seeks to refine current constraints on relative fractions of lower mantle mineralogical constituents. Specifically, the authors employed Brillouin scattering measurements coupled to resistively heated diamond-anvil cells to observe shear wave velocities (vS) in polycrystalline $CaSiO_3$ davemaoite up to about 80 GPa, 973 K. The experimental outcome was employed, together with literature data, to constrain the phase boundary between tetragonal/cubic $CaSiO_3$ polymorphs in a P-T space relevant to the Earth’s mantle, and obtain thermoelastic parameters of interest through inversion. Furthermore, the authors employed their thermoelastic parameters to calculate cubic $CaSiO_3$ properties (vP, vS and density) along a mantle adiabat geotherm, and proceed with a comparison of these results to the Preliminary reference Earth Model (PREM; Dziewonski and Anderson, 1981). Finally, the authors employed their fit results to calculate acoustic velocity at 40 and 100 GPa of lower mantle aggregates having different vol.% of $CaSiO_3$ davemaoite.

According to the authors, their research is broadly impactful since: (a) it provides the first experimental constraints on the vS velocities of $CaSiO_3$ davemaoite at pressures representative of the lower mantle and HT; (b) it shows that $CaSiO_3$ davemaoite in the lower mantle can only be present in its cubic polymorph, including in recycled lithospheric materials (e.g., meta-basalts); (c) it suggest the presence of mineralogical heterogeneities in the lowermost mantle, and provides an alternative perspective on the peculiar seismic characteristics of the Large Low Shear Velocity Provinces (LLSVPs).

First, I would like to congratulate the authors for their experimental results since they managed to perform some rather challenging experiments. However, I am not convinced that the manuscript meets the requirements for publication in Nature Communication and I suggest rejection. This overall decision is based on two main points. Specifically:

• I think that the observations will be of interest to those working on the lower mantle and/or related topics. However, I do not believe that the manuscript provides an advance in our understanding of lower mantle mineralogy having sufficient importance to be published in Nature Communication. This point is further discussed in Comment 1 and 2 below;

• I am not convinced that the experimental dataset supports the authors’ claim. Unfortunately, I think both the experimental approach followed and dataset collected display intrinsic issues, and I am afraid I do not have any suggestion to share with the authors to address them. This point is detailed in Comment 3 and 4 below;

Detailed comments on my first point:

1. As mentioned earlier, I think that the experimental dataset would be valuable for the scientific community since the authors

indeed provide the first experimental dataset on vS velocities of $CaSiO_3$ davemaoite at lower mantle pressures and high temperature. However, davemaoite is expected to incorporate Fe, as well as minor amounts of Mg, Al and Ti, in its crystal structure. In this context, a study targeting the HP/T elasticity of Fe-bearing davemaoite by Brillouin scattering measurements and XRD at HP/T was recently published in Science (Zhou et al., 2025). It is important to note that Zhou et al. explored a wider P-T space (compared to the manuscript presented here); it also produced results with higher accuracy due to the coupling of Brillouin scattering with XRD measurements, and last but not least Zhou et al. investigated a sample with a composition representative for lower mantle lithologies. I am afraid that this limits the potential impact of the proposed manuscript.

2. Regarding the phase boundary, it is true that discrepancies exist among theoretical vs experimental results as stated by the authors (Line 39-44), and this is not surprising, as also pointed out by the authors (line 44-46). At the same time, experimental constraints about the tetragonal-cubic transition in $CaSiO_3$ are available already (Komabayashi et al., 2007), and compositional effects have been (partially) explored too (e.g., Kurashina et al., 2004; Yu et al., 2026). Importantly, the results provided here, although these are questionable (see comment 3), appear to be in rather good agreement with one of the above-mentioned studies, i.e. Komabayashi et al. (2007). I will not comment further on this specific aspect since the authors decided to not compare their results with (Kurashina et al., 2004; Yu et al., 2026). Yet, I am afraid I see the proposed findings to be a confirmation of previous experimental studies, as also indirectly implied by the authors (line 39-42, 87-90). As such, I am afraid but I miss to see the novelty here.

Detailed comments on my second point:

3. From my understanding, the phase transition boundary at P>20 GPa is determined through the interpretation of vS behavior in heating runs at HP (Line 73-81). As shown in Fib1b, vS appear to reach a maximum upon heating, followed up by a rather monotonic decrease upon further heating (Line 75-78). This vS behavior resembles that reported by (Thomson et al., 2019) at ~15 GPa and HT, and is being interpreted by the authors to be characteristic of the transition (Line 78-81). Yet, constraining the phase boundary on the interpretation of vS behavior displayed by the Brillouin scattering dataset presented here is highly problematic because:

3.1. The variation (increase/reduction) in vS magnitude is within uncertainties of individual observations, hence non resolvable, thus effectively preventing a determination of the phase boundary. For example, in their runs at ~65 and ~80 GPa , the authors assigned all observations above 700 K to the cubic phase (Fib1b; Fig 2); However, for example, the largest difference in vS magnitude is < 50 m/s between ~500 K and ~900 K, thereby within uncertainty (see also comment 6 below) of most individual observations (C4P1 and C5P1 in Ext table 1). The same applies to runs at lower pressure. This issue is partially masked by the fact that the authors decided to not report uncertainties in Figure 1b. Additionally, such apparent variations are not necessarily linked to the transition, and they could be explained in terms of:

3.1.1. CPO, as suggested by Extended Figure 2, in which the samples at HP/300 K display velocity variations can be up to 60 m/s along different probing directions (i.e., chi angle; line 366).

3.1.2. relatively low measurement reproducibility, as could be inferred by the data scattering as a function of pressure along a 300 K isotherm (Fig. 1a). Now, this should not come across as a surprise, since one may expect reproducibility to be as good as 0.5% of the measured velocities in single crystals and/or fluids (Sanchez-Valle et al., 2013; Speziale and Duffy, 2002), but reproducibility >0.5% on measured velocities is likely for elastically anisotropic polycrystalline material, hence the need for redundant measurements to obtain accurate results of aggregate properties (e.g., Sinogeikin et al., 2004). Unfortunately, the dataset shows that only a single observation has been collected for each P-T point at T>300K.

3.2. Generally speaking, this approach is based on a rather questionable assumption, being that the material at target has entirely transformed into a crystalline phase prior to Brillouin scattering measurements, and ultimately into the cubic polymorph with resistively heated DAC during Brillouin scattering measurements. The manuscript does not provide any direct/univoque evidence in this regard, in fact, there is a spectral contribution assigned to glass (amorphous $CaSiO_3$, I assume) in selected spectra collected at 80 GPa (Extended Figure 3). This aspect is particularly crucial, because there is possibility that:

3.2.1. the observed peak attributed to the vS of crystalline $CaSiO_3$ might be a superposition of the contributions from grains from both tetragonal and cubic phases especially in the proximity of a transition point, due to kinetics and/or grain interactions (e.g., Wang et al., 2023);

3.2.2. the phase transition might be influenced by the presence of amorphous materials (Extended Figure 3), and two-phase samples can display rather complex compressional behavior (e.g., Glazyrin et al., 2016; Trautner et al., 2026), which are not addressed in the current manuscript;

4. Regarding the implications, the current paper aims at estimating the effect that volumetric abundance of $CaSiO_3$ has on the vS of lower mantle isotropic aggregates at 40 and 100 GPa (and I assume 300 K, since I was not able to find an explicit mention in the manuscript). For each pressure at target, the authors assumed a given composition for bridgmanite (bdm) and ferropericlase (fp). For simplicity (Line 155), the authors also assumed the electronic configuration of Fe^{2+} in fp to be high spin and low spin, at 40 and 100 GPa, respectively. In short, the authors point out that a $CaSiO_3$-bearing aggregate would display lower vS than a $CaSiO_3$ -free aggregate. This is particularly evident at relatively high pressures, prompting the authors to suggest that a normal lower mantle is likely depleted in $CaSiO_3$. Furthermore, the authors suggest that $CaSiO_3$ davemaoite may be concentrated in the LLSVPs, a scenario that could be explained through the crystallization of residual

Ca-rich melts segregated in the deepest regions of the mantle. I am afraid that the evidence supporting these claims are difficult to validate and replicate, mostly due to relatively simplistic approach the authors followed and scarcity of details presented, making the implication section feel highly speculative. For example:

4.1. The authors model Al-bearing bdm. It is unclear if the authors modeled bdm as a multi-component system, and if so, there is no information reported regarding the molar fractions of $FeAlO_3$, $FeFeO_3$ and $AlAlO_3$ components which one may expect to be employed in such modelling. The information reported in extended table 3 is not sufficient to validate their results;

4.2. Calculations are performed at HP/300 K despite the authors having thermoelastic parameters. As such, temperature effects are not considered, despite these being critical (e.g., Deng et al., 2023).

4.3. There is mounting experimental and seismological evidence that fp displays a mixed-spin state across the entire lower mantle and spin fraction varies with depth (e.g., Cobden et al., 2024; Méndez et al., 2022; Trautner et al., 2024, 2023). This effect is not captured by the modelling approach proposed by the authors thereby lowering the overall accuracy of their results;

4.4. The origin and nature of LLSVPs is still highly debated, despite authors' claims (Line 99-101)- on the top of my head for example it has been recently suggested that they might have a thermal origin (Muir et al., 2026);

4.5. The proposed dataset does not provide information on elastic anisotropy, hence the authors are forced to model isotropic aggregates. Therefore, dynamics cannot be considered and/or discussed, yet these are crucial to understand lower(most) mantle seismic signature & mineralogy (e.g., Gay et al., 2024), including LLSVPs (e.g., Deng et al., 2017).

I would like to add a general comment, as well as major and minor corrections. I hope the authors would consider them independently on the venue in which the manuscript will be ultimately published.

General comments:

5. I found several arguments the authors make to be hard to follow and I would like to suggest the authors to improve readability of their manuscript through rephrasing. As an example, Line 27-30, the authors state that "because of direct sampling of the lower mantle is not feasible, our understanding relies primarily on seismic observations, which in turn require robust constraints on the elastic properties of relevant minerals under high-pressure high-temperature conditions". Such statement may be unclear to the reader for the following reasons: a) it is unclear why sampling of the lower mantle is not feasible (I would suggest the authors to either clarify this aspect to the reader, or simply omit this part); b) it is unclear what information can be extracted by seismic observations (e.g., current thermo-physical state of the mantle? Mineralogical, chemical composition of the mantle?); c) the contribution that "robust" constraints on physical properties of mantle minerals may provide to this topic is also unclear (why would these constraints help with observations? I mean, observations remain observations, it's all about their interpretation…). Furthermore, the authors may want to employ a more appropriate language to describe their research, for example: a) I do not necessarily believe that the term robust is appropriate here – the authors could say accurate and precise constraints instead); b) the use of "relevant minerals" cut out all those materials that are not mineral (e.g., phases), yet are present in the lower mantle and may contribute to the seismic signature of the lower(most) mantle. Similarly, I found hard to follow and I suggest rephrasing in:

5.1. Line 31-38, I would suggest the authors to not employ the term progressively since the kinetics of this transition are not well explored in literature and/or in the present study. Also, I think it would be appropriate to say that it would be important to define the boundary of this transition in a P-T space relevant to the lower mantle (line 36-38), which in the end is what the authors do in their research.

5.2. Line 39-49. I think this subsection could benefit from rephrasing, because it starts by saying that ''the phase boundary is poorly constrained'', but then argue that experimental and theoretical results are available. The authors could, for example, remove the statement in line 39, and simply discuss discrepancies between experimental and theoretical studies. Furthermore, I would suggest the authors to clarify and expand their statement regarding "independent experimental constraints" because I find it unclear. Ultimately, I would like to note that the character of this transition in $CaSiO_3$ has yet to be explored, but it may be worth having a look and include in the discussion spontaneous strains studies coupled to Landau theory on tetragonal-cubic transition in perovskites (Carpenter and Salje, 1998; Perks et al., 2014), and of course keep in mind the elastic softening reported in $CaSiO_3$ (Zhang et al., 2025).

Major corrections:

6. I am concerned that vS uncertainties have been underestimated since most, if not all, uncertainties reported are below 1% (Extended Table 1), including in spectra of relatively poor quality (e.g., 50 m/s uncertainty at 81 GPa, 973 K; spectrum in extended figure 3). I would like to note that Brillouin spectra of polycrystalline sample result from a superposition of light scattered from all grains contained within a scattering volume (e.g., Angel et al., 2009; Sinogeikin et al., 2004; Speziale et al., 2014). Due to the relatively large elastic anisotropy of the cubic polymorph, with a vS anisotropy that can be as large as 31% (Zhang et al., 2025), as well as the anisotropy of the photo-elastic coupling intrinsic to Brillouin scattering measurements, one may expect that the observed spectral features attributed to the vS of $CaSiO_3$ would be associated to peaks having a relatively broad, potentially asymmetric shape, especially when collected in a relatively short time as done in this study (10-30 min, line 374). This seems to be indeed the case, as shown in the spectra presented in Extended Figure 3.

All of these aspects should be considered in the estimation of uncertainties; as such, it would be sensible to propagate both statistical uncertainties resulting from the peak fitting, take into account the standard deviation between stokes and anti-stokes spectral contributions, and ultimately propagate the systematic error induced by the scattering angle calibration as well (0.1 deg; line 354). To validate their results, I would like to suggest the authors to disclose the approach followed to: a) carry out the channel-to-velocity conversion; b) uncertainty determination in observed velocities; if scanning amplitude determination was done through FP1 peak fitting in tandem, I would suggest the authors to report at least one spectrum, collected in tandem, in which the quality of FP1 and FP2 spectral contributions can be evaluated.

7. It is unclear if laser-heating, hence crystallization of $CaSiO_3$, was achieved once the DAC was already pre-compressed to the target pressure. The only piece of information I found is that each DAC was pre-compressed to at least 35 GPa (Line 345, Extended Figure 1). Since texturing is induced through compression (Wenk et al., 2004), and cubic $CaSiO_3$ is relatively weak (Immoor et al., 2022), I think it would be important to provide further supporting evidence that no texturing was induced in $CaSiO_3$ prior to Brillouin measurements, that is, DACs were not further compressed after $CaSiO_3$ synthesis, especially NaCl-free DACs. At the moment, the only evidence provided regards 300K measurements on DACs pre-compressed at 29 and 74.5 GPa (Extended figure 2), and the latter was not used for HT measurements I assume (C1P1 is the only cell at 29 GPa in Extended table 1).
I think it is worth checking that none of the DACs contained textured samples before HT runs, since each sample may be different in term of texture and grain size. Therefore, I suggest the authors replicate what done for C2P1, that is, I suggest the authors to provide an angular dispersion curve of vS at 300 K for each one of the DAC that was later employed for HT runs.

Minor corrections

8. Figure 1a: I suggest the authors to report their fit of velocity data in Figure 1a, not just their observations; This would give the reader a direct mean to visualize potential discrepancies with previous experimental studies as currently discussed in Line 120-123.

9. I suggest the authors to clarify the correction applied to high-P/T velocity data to validate their results; I further suggest the authors to report both non-corrected and corrected values as supplementary material.

10. Line 77-81, in fact, one may expect a decrease of G in the proximity of the transition (Zhang et al., 2025) , which should be reflected in a local minimum of vS at the transition point. Elastic softening may also be present in the cubic phase (Zhang et al., 2025);

11. Line 82-83, this is obvious since the phase boundary was determined in combination with results from ultrasonic measurements as well; I suggest the authors to remove this statement.

12. Line 94-96, I suggest the authors to further clarify that this is a confirmation of previous XRD, as previously stated in line 39-42;

13. Line 97-101, the nature of LLSVP and ULVZ is highly debated (McNamara, 2019), and more recently (Muir et al., 2026). I suggest the authors to improve the current discussion on the state-of-the-art regarding LLSVP and ULVZ so to place the manuscript in a more updated context.

14. Line 101-108, I do not agree with this statement. From my understanding, the phase boundary by (Zhang et al., 2025) does not intersect the normal mantle geotherm at the base of the lower mantle, in fact, it is even below the cold slab geotherm, see their Figure 2. I suggest them to clarify their statement.

15. Line 368-369 I do not necessarily agree that cubic $CaSiO_3$ is nearly elastically isotropic, since it contrasts with recent literature (Zhang et al., 2025). I suggest the expand this aspect through discussion.

References:
Angel, R.J., Jackson, J.M., Reichmann, H.J., Speziale, S., 2009. Elasticity measurements on minerals: a review. European Journal of Mineralogy 21, 525–550. https://doi.org/10.1127/0935-1221/2009/0021-1925
Carpenter, M.A., Salje, E.K.H., 1998. Elastic anomalies in minerals due to structural phase transitions. ejm 10, 693–812. https://doi.org/10.1127/ejm/10/4/0693
Cobden, L., Zhuang, J., Lei, W., Wentzcovitch, R., Trampert, J., Tromp, J., 2024. Full-waveform tomography reveals iron spin crossover in Earth's lower mantle. Nat Commun 15, 1961. https://doi.org/10.1038/s41467-024-46040-1
Deng, J., Long, M.D., Creasy, N., Wagner, L., Beck, S., Zandt, G., Tavera, H., Minaya, E., 2017. Lowermost mantle anisotropy near the eastern edge of the Pacific LLSVP: constraints from SKS–SKKS splitting intensity measurements. Geophysical Journal International 210, 774–786. https://doi.org/10.1093/gji/ggx190
Deng, X., Xu, Y., Hao, S., Ruan, Y., Zhao, Y., Wang, W., Ni, S., Wu, Z., 2023. Compositional and thermal state of the lower mantle from joint 3D inversion with seismic tomography and mineral elasticity. Proceedings of the National Academy of Sciences 120, e2220178120. https://doi.org/10.1073/pnas.2220178120
Dziewonski, A.M., Anderson, D.L., 1981. Preliminary reference Earth model. Physics of the Earth and Planetary Interiors 25, 297–356. https://doi.org/10.1016/0031-9201(81)90046-7
Gay, J.P., Ledoux, E.E., Krug, M., Chantel, J., Pakhomova, A., Sanchez-Valle, C., Speziale, S., Merkel, S., 2024. Depth Dependent Deformation and Anisotropy of Pyrolite in the Earth's Lower Mantle. Geophysical Research Letters 51, e2024GL109433. https://doi.org/10.1029/2024GL109433
Glazyrin, K., Miyajima, N., Smith, J.S., Lee, K.K.M., 2016. Compression of a multiphase mantle assemblage: Effects of

undesirable stress and stress annealing on the iron spin state crossover in ferropericlase. Journal of Geophysical Research: Solid Earth 121, 3377–3392. https://doi.org/10.1002/2015JB012321
Immoor, J., Miyagi, L., Liermann, H.-P., Speziale, S., Schulze, K., Buchen, J., Kurnosov, A., Marquardt, H., 2022. Weak cubic CaSiO3 perovskite in the Earth's mantle. Nature 603, 276–279. https://doi.org/10.1038/s41586-021-04378-2
Komabayashi, T., Hirose, K., Sata, N., Ohishi, Y., Dubrovinsky, L.S., 2007. Phase transition in CaSiO3 perovskite. Earth and Planetary Science Letters 260, 564–569. https://doi.org/10.1016/j.epsl.2007.06.015
Kurashina, T., Hirose, K., Ono, S., Sata, N., Ohishi, Y., 2004. Phase transition in Al-bearing CaSiO3 perovskite: implications for seismic discontinuities in the lower mantle. Physics of the Earth and Planetary Interiors 145, 67–74. https://doi.org/10.1016/j.pepi.2004.02.005
McNamara, A.K., 2019. A review of large low shear velocity provinces and ultra low velocity zones. Tectonophysics 760, 199–220. https://doi.org/10.1016/j.tecto.2018.04.015
Méndez, A.S.J., Stackhouse, S., Trautner, V., Wang, B., Satta, N., Kurnosov, A., Husband, R.J., Glazyrin, K., Liermann, H.-P., Marquardt, H., 2022. Broad Elastic Softening of (Mg,Fe)O Ferropericlase Across the Iron Spin Crossover and a Mixed-Spin Lower Mantle. Journal of Geophysical Research: Solid Earth 127, e2021JB023832. https://doi.org/10.1029/2021JB023832
Muir, J.M.R., Chen, Q., Zhang, F., 2026. A Thermal Origin of LLSVP Seismic Signals? Consequences of Perovskite Phase Mixing in the Deep Lower Mantle. Journal of Geophysical Research: Solid Earth 131, e2025JB033647. https://doi.org/10.1029/2025JB033647
Perks, N.J., Zhang, Z., Harrison, R.J., Carpenter, M.A., 2014. Strain relaxation mechanisms of elastic softening and twin wall freezing associated with structural phase transitions in (Ca,Sr)TiO3 perovskites. J. Phys.: Condens. Matter 26, 505402. https://doi.org/10.1088/0953-8984/26/50/505402
Sanchez-Valle, C., Mantegazzi, D., Bass, J.D., Reusser, E., 2013. Equation of state, refractive index and polarizability of compressed water to 7 GPa and 673 K. J. Chem. Phys. 138, 054505. https://doi.org/10.1063/1.4789359
Sinogeikin, S.V., Zhang, J., Bass, J.D., 2004. Elasticity of single crystal and polycrystalline MgSiO3 perovskite by Brillouin spectroscopy. Geophysical Research Letters 31. https://doi.org/10.1029/2004GL019559
Speziale, S., Duffy, T.S., 2002. Single-crystal elastic constants of fluorite (CaF2) to 9.3 GPa. Phys Chem Min 29, 465–472. https://doi.org/10.1007/s00269-002-0250-x
Speziale, S., Marquardt, H., Duffy, T.S., 2014. Brillouin Scattering and its Application in Geosciences. Reviews in Mineralogy and Geochemistry 78, 543–603. https://doi.org/10.2138/rmg.2014.78.14
Thomson, A.R., Crichton, W.A., Brodholt, J.P., Wood, I.G., Siersch, N.C., Muir, J.M.R., Dobson, D.P., Hunt, S.A., 2019. Seismic velocities of CaSiO3 perovskite can explain LLSVPs in Earth's lower mantle. Nature 572, 643–647. https://doi.org/10.1038/s41586-019-1483-x
Trautner, V.E., Koemets, E., Wang, B., Satta, N., Buchen, J., Hughes, G.M., Ledoux, E.E., Marquardt, K., Fei, H., Giordano, N., Katsura, T., Marquardt, H., 2026. Shielding of periclase by bridgmanite during compression of a polycrystalline mantle assemblage. American Mineralogist. https://doi.org/10.2138/am-2025-10040
Trautner, V.E., Rijal, A., Plueckthun, C., Satta, N., Koemets, E., Buchen, J., Wang, B., Glazyrin, K., Cobden, L., Marquardt, H., 2024. Iron Content-Dependence of Ferropericlase Elastic Properties Across the Spin Crossover From Novel Experiments and Machine Learning. Geophysical Research Letters 51, e2024GL111276. https://doi.org/10.1029/2024GL111276
Trautner, V.E., Stackhouse, S., Turner, A.R., Koelemeijer, P., Davies, D.R., Méndez, A.S.J., Satta, N., Kurnosov, A., Liermann, H.-P., Marquardt, H., 2023. Compressibility of ferropericlase at high-temperature: Evidence for the iron spin crossover in seismic tomography. Earth and Planetary Science Letters 618, 118296. https://doi.org/10.1016/j.epsl.2023.118296
Wang, B., Buchen, J., Méndez, A.S.J., Kurnosov, A., Criniti, G., Liermann, H.-P., Marquardt, H., 2023. Strong Effect of Stress on the Seismic Signature of the Post-Stishovite Phase Transition in the Earth's Lower Mantle. Geophysical Research Letters 50, e2023GL102740. https://doi.org/10.1029/2023GL102740
Wenk, H.-R., Lonardeli, I., Pehl, J., Devine, J., Prakapenka, V., Shen, G., Mao, H.-K., 2004. In situ observation of texture development in olivine, ringwoodite, magnesiowüstite and silicate perovskite at high pressure. Earth and Planetary Science Letters 226, 507–519. https://doi.org/10.1016/j.epsl.2004.07.033
Yu, Y., Zhang, X., Zhang, D., Li, L., Mao, Z., Sun, N., Wang, D., Li, J., Zhao, C., Qian, C., Wei, Y., Li, X., Wang, Y., Ishii, T., 2026. Davemaoite Elasticity Reveals Slab-Induced Heterogeneity in the Mantle Transition Zone. Geophysical Research Letters 53, e2025GL118147. https://doi.org/10.1029/2025GL118147
Zhang, C., Yang, J.-Y., Sun, T., Zhang, H., Brodholt, J.P., 2025. Strong precursor softening in cubic CaSiO3 perovskite. Proceedings of the National Academy of Sciences 122, e2410910122. https://doi.org/10.1073/pnas.2410910122
Zhou, W.-Y., Hao, M., Su, W., Kim, T., Chen, S., Shim, S.-H., Zhang, D., Nguyen, P.Q.H., Armstrong, K., Zhang, J.S., 2025. Elasticity of davemaoite as a primary contributor to lower-mantle heterogeneities. Science 390, 935–939. https://doi.org/10.1126/science.adx8356

Version 1:

Reviewer comments:

Reviewer #1

(Remarks to the Author)
The authors have addressed my comments.

Reviewer #2

(Remarks to the Author)

The authors have adequately addressed the reviewers' comments and revised the manuscript accordingly. The responses provided in the rebuttal letter are clear and satisfactory, and the revisions have improved the quality and clarity of the manuscript. I have no further concerns and recommend acceptance for publication.

Reviewer #4

(Remarks to the Author)

The manuscript by Hao et al. presents new high-pressure and high-temperature Brillouin spectroscopy measurements of $CaSiO_3$ davemaoite up to 80 GPa and 973 K. By combining these new elasticity measurements with previously published volume data, the authors derive an updated thermoelastic model for cubic davemaoite and discuss its implications for lower-mantle mineralogy and the origin of Large Low Shear Velocity Provinces (LLSVPs).
The manuscript addresses an important problem in mineral physics and deep-Earth geophysics. Reliable sound velocity measurements under true lower-mantle pressure–temperature conditions are essential not only for constraining the elastic properties and mineralogy of the Earth's lower mantle, but also because they provide one of the few experimental probes capable of detecting structural phase transitions that are difficult to resolve using conventional X-ray diffraction. This is particularly relevant for the tetragonal-to-cubic transition in $CaSiO_3$ davemaoite, where the structural distortion is subtle and diffraction peak splitting becomes increasingly difficult to resolve at high pressures and temperatures. In this respect, high-pressure Brillouin spectroscopy represents a powerful complementary technique, and the present study has the potential to provide an important contribution to our understanding of both the phase transition and the thermoelastic properties of davemaoite under lower-mantle conditions.
Overall, I find the work technically impressive, well executed, and potentially suitable for publication in Nature Communications. However, several important issues should be addressed before the conclusions can be fully supported. In particular, the interpretation of the observed temperature dependence of the shear-wave velocity as direct evidence for the tetragonal-to-cubic phase transition requires additional justification. I therefore recommend major revision.
Major Comment
A major strength of this study is the use of high-pressure high-tempearature Brillouin spectroscopy as an alternative probe for detecting the tetragonal-to-cubic phase transition in davemaoite, which is difficult to resolve by X-ray diffraction under lower-mantle conditions. Because this interpretation forms the basis of several key conclusions of the manuscript, it is essential to demonstrate convincingly that the observed temperature dependence of the shear-wave velocity is uniquely associated with the phase transition and cannot arise from experimental artefacts.
The authors interpret the observed increase of the shear-wave velocity during the initial stage of heating, followed by a decrease above a characteristic temperature, as the transition from tetragonal to cubic davemaoite. While this interpretation is plausible and is consistent with previous ultrasonic studies, an increase of shear-wave velocity with increasing temperature is nevertheless unusual for perovskite-type materials. Therefore, alternative explanations should be carefully excluded.
During the experiments, pressure was determined from the Raman edge of the diamond anvils, whereas temperature was monitored by thermocouples attached to the gasket. Both pressure and temperature influence the Raman signal of diamond, and pressure variations associated with thermal expansion of the diamond anvil cell during resistive heating are well known. I appreciate that the authors performed experiments using anvils mounted on seats with different thermal expansion coefficients and provide a careful discussion of pressure uncertainties in the Supplementary Information. Nevertheless, I believe that additional experimental evidence would considerably strengthen the manuscript.
In particular, I encourage the authors to demonstrate that the anomalous temperature dependence is unique to davemaoite. Whenever possible, Brillouin peaks originating from the residual glass and/or the pressure-transmitting medium should be presented and compared with the behaviour of the davemaoite peak under identical experimental conditions. If these peaks exhibit the expected monotonic decrease in velocity with increasing temperature while only davemaoite displays the initial increase in velocity, this would provide compelling evidence that the observed behaviour is intrinsic to the sample rather than an artefact related to pressure determination or pressure-temperature correlations in the Raman calibration.
In addition, the manuscript would benefit from a more comprehensive discussion of the possible physical origin of the positive temperature dependence of the shear-wave velocity in tetragonal davemaoite. The manuscript provides only a brief discussion of the underlying mechanism. Expanding this discussion and placing the observations into the broader context of previous experimental and theoretical studies would strengthen the interpretation considerably.
Minor Comments
1. Line 51: The phrase "absolute elastic properties" is unclear. Do the authors refer to discrepancies between experiments and theoretical predictions?
2. Lines 77–79: The wording "progressive transformation" is thermodynamically ambiguous. Strictly speaking, a phase transition itself is not progressive. If the transition extends over a finite temperature interval, this should reflect phase coexistence resulting from kinetic effects, pressure gradients, or temperature gradients within the sample. I suggest rephrasing this section accordingly.
3. Line 79: The sentence "Once temperatures exceed the phase boundary..." assumes the interpretation before presenting the observations. It would be more objective first to describe the experimental observation (e.g., "Above a certain temperature, the measured shear-wave velocity decreases monotonically...") and only then discuss its interpretation in terms of the phase transition.
4. Line 247: "...davemaoite is supposed to by tetragonal..." → "...supposed to be tetragonal..."
5. Methods: The observation that the thermocouple reading increases by 1–2 °C demonstrates the sensitivity of the thermocouple but does not necessarily reflect the actual sample temperature. Did the authors monitor the thermocouple response during CO_2-laser heating used for sample synthesis? Conversely, the observed sensitivity may also indicate a certain temperature increase induced by the probing laser itself. Although this would likely not affect the conclusions because identical laser power was used throughout the Brillouin measurements, it may be worth mentioning as a

consideration for future experiments.
6. Supplementary Fig. S5: I could not find any reference to this figure in either the main text or the Supplementary Information, and its purpose is not clear. The figure appears to show a standard Fabry–Pérot calibration spectrum acquired in alignment mode, where interaction between FP1 and FP2 is not expected. Rather than including this figure, I suggest that the authors provide a brief description of the scattering-angle calibration procedure. The Supplementary Information states that an angular uncertainty of 0.1° contributes only ~10 m/s to the velocity uncertainty, but the basis for this estimate is not explained. A short description of the calibration procedure would improve the transparency and reproducibility of the experimental methodology. Since this uncertainty is systematic, it does not affect the observed temperature-dependent trends or the qualitative conclusions of the study; however, it contributes to the uncertainty of the absolute sound velocities and, consequently, to the derived thermoelastic parameters.
Overall, this is an interesting and potentially impactful study. I believe it is suitable for publication in Nature Communications after the authors have satisfactorily addressed the points raised above.

Version 2:

Reviewer comments:

Reviewer #4

(Remarks to the Author)
The authors have addressed all of my comments satisfactorily. I appreciate the thorough revisions and the additional clarifications provided in both the manuscript and the response letter. I have no further concerns and believe that the manuscript is now suitable for publication in Nature Communications.

Open Access This Peer Review File is licensed under a Creative Commons Attribution 4.0 International License, which permits use, sharing, adaptation, distribution and reproduction in any medium or format, as long as you give appropriate credit to the original author(s) and the source, provide a link to the Creative Commons license, and indicate if changes were made.
In cases where reviewers are anonymous, credit should be given to 'Anonymous Referee' and the source.
The images or other third party material in this Peer Review File are included in the article’s Creative Commons license, unless indicated otherwise in a credit line to the material. If material is not included in the article’s Creative Commons license and your intended use is not permitted by statutory regulation or exceeds the permitted use, you will need to obtain permission directly from the copyright holder.
To view a copy of this license, visit https://creativecommons.org/licenses/by/4.0/

Response to the Reviewers

We thank the three reviewers for their comments. The detailed responses to each of the Reviewer's comments are listed below (their points are in blue; our answers are in black). All revisions made to the manuscript and Supplementary Materials are highlighted in blue, and revised figures are indicated by blue titles.

Response to comments by Reviewer #1:

Reviewer #1 (Remarks to the Author):

Hao et al measure the elastic properties and P-T slope second-order cubic-tetragonal transition in CaSiO3 perovskite (Davemaoite) to 80 GPa and 973 K using Brillouin spectroscopy in a resistively heated diamond anvil cell. Their estimates of the P-T slope and position of the transition are consistent with lower pressure (including larger volume) measurements.

Uncertainties in the cubic-tetragonal transition in CaSiO3 have plagued deep Earth geophysics for decades, and this study more or less puts the question to rest: At plausible lower mantle P-T conditions, only the cubic phase appears to stable. Of course there are always some lingering caveats. For example, DACS are plagued by deviatoric stresses, may be sensitive to deviatoric stress owing to its expression as a lattice distortion. However, I think the present experimental approach is the best one can hope for to mitigate most issues.

Less certain is the extrapolation of elastic properties to higher temperatures (well above the experimental conditions), however this is a longstanding issue that relates to technical limitations of the Brillouin spectroscopy approach. Nevertheless, the authors have admitted to generous uncertainties and I do not find their estimates to be over-stated beyond the support of the underlying data. In terms of known-unknowns, I would consider the spin transition in ferropericlase to be a significant source of uncertainty, particularly its potential influence upon Fe partitioning.

The interpretation of LLSVP as cubic Ca-Pv (davemaoite)-rich following fractionation of a basal magma ocean is plausible, as it is last to crystallize from any plausible starting composition. The depletion of the remainder of the lower mantle in CaO is also possible, although it also requires some underlying rationale such as containing cumulates of the magma ocean and/or the BEAMS hypothesis of Ballmer et al. (2017). In any case I think the lower mantle chemical composition is poorly constrained and I welcome ideas such as those presented in this paper.

There is only one minor point that I would raise, which is the claim that LLSVP comprise ~8 vol% of the mantle. In fact, the cited study does not aim to measure LLSVP alone, but rather a broader class of lower velocity anomalies. Instead, the vast majority of studies aiming to constrain LLSVP volume specifically (my recent student catalogued these to compare with his

own estimate) find it to be only 2-3% of the total mantle (similar to continents). The LLSVP appear large in map view, however, it is important to note that the CMB is ~4x smaller in area than Earth's surface (accordingly, LLSVP are estimated to be roughly ~3-4x thicker than continents, extending ~600-800 km above the CMB at their peaks).

Sincerely,
John Hernlund
(Nozomi 163, 15 March 2026)

We sincerely thank John Hernlund for his thoughtful and supportive comments, as well as for recognizing the contribution of our work to the broader research community. In response, we have revised the discussion in Line 194-196 to more clearly reflect the complexity of LLSVPs and the uncertainties associated with their volumetric estimates.

Response to comments by Reviewer #2:

Reviewer #2 (Remarks to the Author):

This manuscript presents new high-pressure and high-temperature Brillouin spectroscopy measurements on $CaSiO_3$ davemaoite, providing important constraints on the tetragonal-cubic phase transition and its elastic properties under lower mantle conditions. By combining these new data with previous experimental results, the authors refine the phase boundary and derive updated thermoelastic parameters, which have significant implications for interpreting seismic observations and understanding lower mantle mineralogy and heterogeneity. In particular, the conclusion that tetragonal davemaoite is unlikely to be stable along any plausible mantle geotherm, and the suggestion that davemaoite may be depleted in the ambient lower mantle but enriched in LLSVPs, are both scientifically meaningful and relevant to current debates in deep Earth geophysics.
Overall, the study is of clear scientific value and suitable for publication. However, there are several points that require clarification or further discussion. Addressing these issues would improve the rigor and clarity of the manuscript. I therefore recommend acceptance after revision.

We thank reviewer #2 for the supportive comments and suggestions.

Specific comments
1. The tetragonal and cubic perovskite structures of $CaSiO_3$ are very similar, and their XRD patterns differ only subtly. In Extended Data Fig. 1, the XRD pattern was collected at ~43 GPa, but the temperature is not specified. I assume that this measurement was performed at room temperature. If so, $CaSiO_3$ should adopt the tetragonal perovskite structure under these conditions. However, all diffraction peaks in the figure are indexed as belonging to the cubic

structure, which may not be appropriate. The authors are encouraged to clarify the experimental temperature and justify the structural assignment.

We thank the reviewer for highlighting the possibility of refining the structure of davemaoite using the XRD data. We attempted to fit the XRD patterns using both cubic and tetragonal models; however, the resulting fits are very similar and do not allow for a clear distinction between the two structures. Owing to the experimental setup at the beamline (in particular the X-ray wavelength and the sample–detector distance), the structural differences cannot be resolved. This limitation is consistent with that reported in a recent study (Zhou et al., 2025). We have added this clarification to Line 246-248.

2. The authors determine the phase transition based on the temperature dependence of Vs shown in Fig. 1(b), which is consistent with approaches used in previous studies. However, I wonder whether the authors have collected XRD patterns for the sample both before and after the phase transition. Is it possible to directly identify the phase transition from XRD data? Although the structural differences between tetragonal and cubic CaSiO3 are subtle, the tetragonal phase may exhibit peak splitting and weak reflections compared to the cubic phase. Any additional structural evidence from XRD would strengthen the conclusions.

We thank the reviewer for the helpful suggestions. As noted above, even at 300 K, the resolution achievable at the current beamline does not allow us to distinguish between the possible structures of davemaoite. In addition, our external heating system—including the heaters and vacuum enclosure—is specifically designed for Brillouin measurements in our laboratory and is not compatible with the cell holders used at synchrotron facilities. In future work, we may explore the possibility of collaborating with beamline scientists to assess the feasibility of conducting high-temperature experiments.

Furthermore, XRD has already been employed to investigate the phase transition of pure $CaSiO_3$ davemaoite in a previous study (Komabayashi et al., 2007). Rather than duplicating this work, our study aims to apply complementary approaches to help resolve the discrepancies between experimentally constrained phase boundaries and those predicted by theoretical calculations.

3. The pressure in the DAC experiments was determined using the Raman edge of diamond anvils. This method may introduce relatively large uncertainties at low pressures, depending on the laser focus position within the sample chamber and its depth relative to the diamond culet surface. In addition, during heating at high pressure, thermal pressure can develop, causing the pressure to increase with temperature. Although the authors mention that pressure corrections were applied during heating, it is not entirely clear how uncertainties arising from (i) Raman pressure calibration and (ii) thermal pressure effects were treated. Were these uncertainties explicitly incorporated into the further high-pressure fitting (third-order Birch-Murnaghan and Eulerian finite-strain EoS)? If so, how significant is their impact on the fitted parameters and final conclusions? A more detailed explanation would be helpful.

We thank the reviewer for pointing out the confusion regarding the refinement of pressure uncertainties. Accurate pressure determination at high temperatures is indeed critical. During the setup of our system, we evaluated the accuracy of diamond anvil Raman measurements at relatively low pressures. As shown in Fig. R1, the diamond Raman spectra at 20–30 GPa exhibit a well-defined edge. With increasing temperature, the signal-to-noise ratio does not show a significant degradation, indicating that the diamond Raman edge remains a reliable method for pressure determination under high-temperature conditions. We are currently preparing a separate manuscript that will present a more detailed discussion of pressure measurements at elevated temperatures.

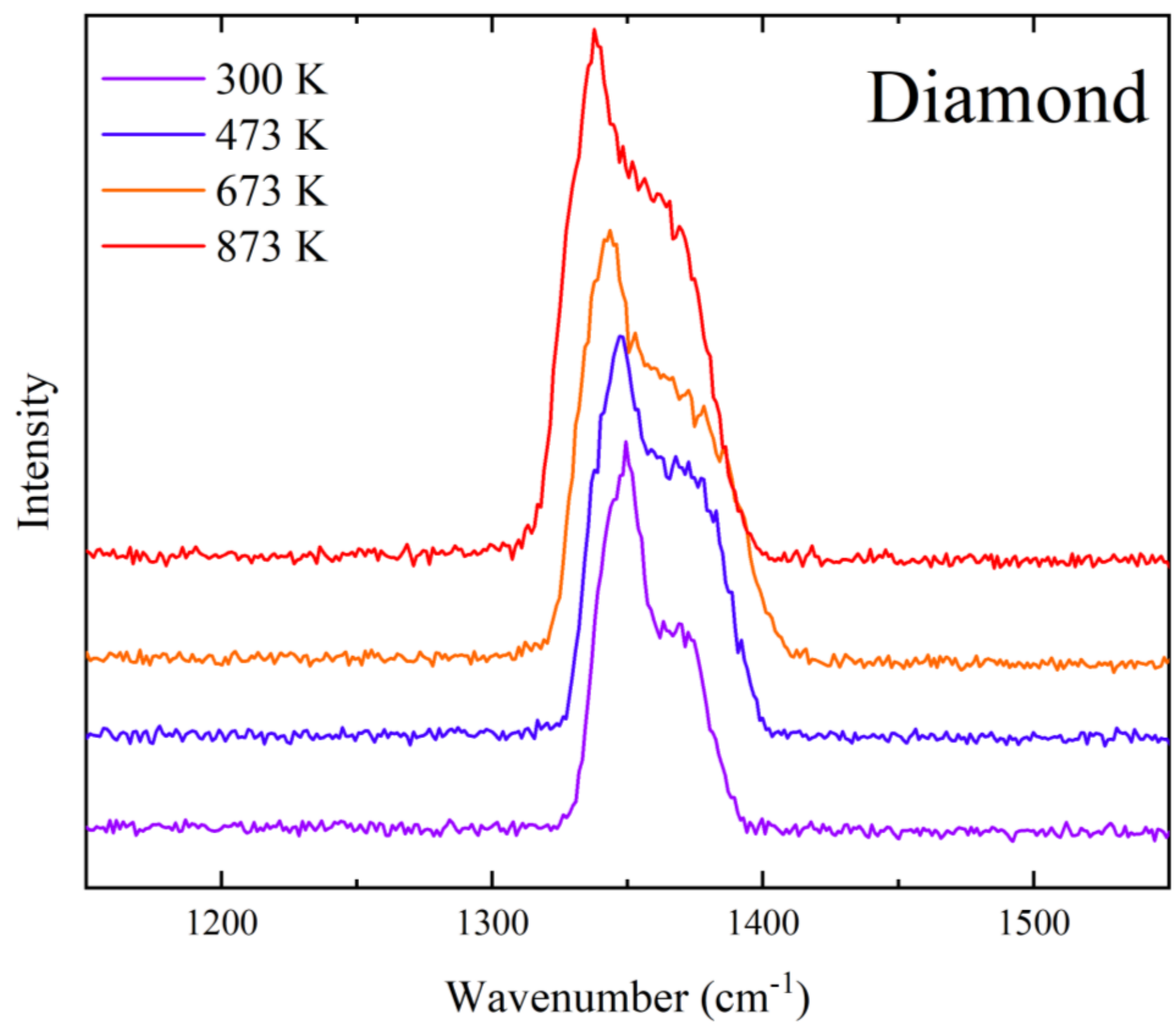

Fig. R1. Typical spectra of diamond anvil Raman gauge at 20-30 GPa under different temperatures with collecting time of 3 s.

As the reviewer noted, the focus and positioning of the laser can influence pressure measurements. In this study, we performed in-situ Raman measurements using the laser spot as the Brillouin. Therefore, when the Brillouin signal is well focused on the sample, the Raman measurements are also well aligned. As a result, the measured pressure directly corresponds to the location from which the Brillouin spectra are collected, minimizing uncertainties associated with pressure gradients within the sample chamber. We have clarified this point Supplementary Materials Text S1.

With regard to thermal pressure, we consider it unnecessary to apply an additional correction for externally resistive heating. During high-temperature experiments, the entire diamond anvils are heated, and pressures are measured directly from the hot anvils using calibrations established under comparable conditions (Ono et al., 2014). As such, thermal effects are inherently accounted for in the measured pressures. In contrast, thermal pressure is typically more significant in laser-heating experiments, where heating is localized and temperature gradients develop within the sample chamber. In such cases, pressures are often determined at room temperature, and a thermal pressure correction is therefore required. We have clarified this point in Supplementary Materials Text S1.

The uncertainties in pressure primarily arise from the fitting of the diamond Raman edge and the coupled effects of temperature. The Raman edge position is temperature dependent, and temperature uncertainties measured by thermocouples are typically on the order of 10–20 K for temperatures up to ~1000 K. These combined effects result in an estimated pressure uncertainty of ~0.2–0.3 GPa at ~900 K. In addition, the limited spectral resolution of our in-situ Raman system (~1 cm^{-1}) introduces fitting uncertainties of ~0.4–0.5 GPa. Consequently, as summarized in Table S1, the overall pressure uncertainty for most high-temperature measurements is ~0.5 GPa. For experiments conducted at higher pressures and temperatures, non-ideal diamond conditions can further increase the fitting uncertainty to ~1–2 GPa. In contrast, room-temperature pressures were measured using a higher-resolution Raman system (~0.3 cm^{-1}) with a smaller focal spot (3–4 μm), resulting in reduced fitting uncertainties of ~0.2–0.5 GPa. We have added these clarifications in Supplementary Materials Text S1.

To evaluate the influence of pressure uncertainties on the fitting results for cubic davemaoite, we performed an additional test in which all pressures were reduced by one standard deviation prior to refitting. This represents an extreme scenario, as the uncertainties would be expected to be more evenly distributed rather than systematically biased toward lower values. The fitting gives K_{t0} = 243(4) GPa, K_{t0}' = 4.09(8), G_0 = 118(2) GPa, G_0' = 1.45(2), θ_0 = 1450(300) K, γ_0 = 2.0(2), q_0 = 1.1(4), and η_0 = 2.5(13). Compared with the values reported in the main text, only G_0' is marginally larger (1.45 VS 1.44) and η_0 slightly smaller (2.5 VS 2.8), both well within their respective uncertainties. These results indicate that the effects of pressure uncertainties are adequately captured within the fitting uncertainties and have a negligible impact on the overall conclusions. We have added the refitting in Supplementary Materials Text S1.

4. In Fig. 2(b), the phase boundary labeled as "Sun et al. (2016)" may be misleading. That study did not explicitly define a phase boundary, but rather showed that CaSiO3 is cubic above ~1200 K. Therefore, including this line could cause confusion, and I suggest removing it.

We thank the reviewer for pointing out the confusion and have revised the figure accordingly.

5. In Fig. 3(b), the authors present Vp and Vs of cubic CaSiO3 at 1500 K and 2500 K, along with extrapolated results from previous low-pressure studies. There are noticeable discrepancies between the present results and previous extrapolations for both Vp and Vs. What is the origin of these differences? The manuscript does not seem to provide sufficient discussion on this point. It

would be useful for the authors to elaborate on the reasons for these discrepancies, such as differences in pressure range, fitting strategy, or thermoelastic parameters.

We thank the reviewer for highlighting the importance of clarifying the discrepancies among different studies. Compared with previous low-pressure ultrasonic measurements (Gréaux et al., 2019; Thomson et al., 2019), our fitted parameters for cubic $CaSiO_3$ davemaoite yield intermediate values for G_0 and its temperature dependence. As ultrasonic techniques fall outside our primary area of expertise, we refrain from assessing the relative reliability of these studies. However, we note several factors that may contribute to the observed differences.

In contrast to Brillouin spectroscopy, ultrasonic measurements are highly sensitive to sample thickness, and uncertainties in thickness determination may introduce additional errors beyond those associated with wave velocity measurements. Furthermore, deformation during crystallization and subsequent heating may alter the wave path and reflection geometry, potentially increasing measurement uncertainty.

The most notable difference between our results and previous ultrasonic studies is the smaller pressure dependence of G observed in our data. This discrepancy may reflect the more limited pressure range explored in ultrasonic experiments (~10 GPa for Gréaux et al., 2019; <5 GPa for Thomson et al., 2019). We have added further clarification in Line 125-129.

<u>Response to comments by Reviewer #3:</u>

Reviewer #3 (Remarks to the Author):

Hao and co-authors present a manuscript that primarily seeks to refine current constraints on relative fractions of lower mantle mineralogical constituents. Specifically, the authors employed Brillouin scattering measurements coupled to resistively heated diamond-anvil cells to observe shear wave velocities (vS) in polycrystalline CaSiO3 davemaoite up to about 80 GPa, 973 K. The experimental outcome was employed, together with literature data, to constrain the phase boundary between tetragonal/cubic CaSiO3 polymorphs in a P-T space relevant to the Earth's mantle, and obtain thermoelastic parameters of interest through inversion. Furthermore, the authors employed their thermoelastic parameters to calculate cubic CaSiO3 properties (vP, vS and density) along a mantle adiabat geotherm, and proceed with a comparison of these results to the Preliminary reference Earth Model (PREM; Dziewonski and Anderson, 1981). Finally, the authors employed their fit results to calculate acoustic velocity at 40 and 100 GPa of lower mantle aggregates having different vol.% of CaSiO3 davemaoite.

According to the authors, their research is broadly impactful since: (a) it provides the first experimental constraints on the vS velocities of CaSiO3 davemaoite at pressures representative

of the lower mantle and HT; (b) it shows that CaSiO3 davemaoite in the lower mantle can only be present in its cubic polymorph, including in recycled lithospheric materials (e.g., meta-basalts); (c) it suggest the presence of mineralogical heterogeneities in the lowermost mantle, and provides an alternative perspective on the peculiar seismic characteristics of the Large Low Shear Velocity Provinces (LLSVPs).

First, I would like to congratulate the authors for their experimental results since they managed to perform some rather challenging experiments. However, I am not convinced that the manuscript meets the requirements for publication in Nature Communication and I suggest rejection. This overall decision is based on two main points. Specifically:

• I think that the observations will be of interest to those working on the lower mantle and/or related topics. However, I do not believe that the manuscript provides an advance in our understanding of lower mantle mineralogy having sufficient importance to be published in Nature Communication. This point is further discussed in Comment 1 and 2 below;

• I am not convinced that the experimental dataset supports the authors' claim. Unfortunately, I think both the experimental approach followed and dataset collected display intrinsic issues, and I am afraid I do not have any suggestion to share with the authors to address them. This point is detailed in Comment 3 and 4 below;

We thank the reviewer for acknowledging the challenges associated with high-temperature experiments at lower-mantle pressures. However, we believe there may be some misunderstanding regarding the novelty and experimental details of our study. We hope that the clarifications provided below will help to address these points.

Detailed comments on my first point:

1. As mentioned earlier, I think that the experimental dataset would be valuable for the scientific community since the authors indeed provide the first experimental dataset on vS velocities of CaSiO3 davemaoite at lower mantle pressures and high temperature. However, davemaoite is expected to incorporate Fe, as well as minor amounts of Mg, Al and Ti, in its crystal structure. In this context, a study targeting the HP/T elasticity of Fe-bearing davemaoite by Brillouin scattering measurements and XRD at HP/T was recently published in Science (Zhou et al., 2025). It is important to note that Zhou et al. explored a wider P-T space (compared to the manuscript presented here); it also produced results with higher accuracy due to the coupling of Brillouin scattering with XRD measurements, and last but not least Zhou et al. investigated a sample with a composition representative for lower mantle lithologies. I am afraid that this limits the potential impact of the proposed manuscript.

We thank the reviewer for recognizing the value of our dataset. However, we believe the concern that our study largely repeats the work of Zhou et al. (2025) may arise from some

misunderstandings regarding the scope and objectives of the two studies. Notably, the first author of the present study is also a coauthor of Zhou et al. (2025), which provides us with a clear understanding of the distinctions between the two works. We therefore would like to clarify several key differences below.

(1) The primary objectives of the two studies differ significantly. Zhou et al. (2025) focused on a compositionally complex davemaoite representative of subducted basaltic crust, which may not be representative of the ambient lower mantle. Accordingly, their interpretations are primarily relevant to slab-related lithologies. In contrast, our study targets davemaoite compositions closer to the $CaSiO_3$ endmember, which are more appropriate for constraining the properties of the ambient mantle. Notably, the sound velocities reported by Zhou et al. (2025) are substantially lower than those of the pure $CaSiO_3$ endmember, making direct application to the broader lower mantle less straightforward.

(2) The experimental approaches also differ in important ways. Zhou et al. (2025) employed laser heating, which can introduce significant temperature gradients and uncertainties, limiting the ability to precisely constrain temperature-dependent elastic properties and phase boundaries. In contrast, our study uses external resistive heating with more stable and well-constrained temperature conditions. This approach enables us to resolve sound velocity changes across the phase transition and to better constrain the cubic–tetragonal boundary of davemaoite at lower-mantle pressures. To our knowledge, this represents the first experimental dataset capturing shear-wave velocity (V_s) variations through this transition under such conditions.

(3) While Zhou et al. (2025) provides valuable insights into Fe-, Mg-, and Al-bearing davemaoite, it remains essential to establish robust constraints on the endmember $CaSiO_3$ system. Such baseline properties are critical for isolating and quantifying the effects of minor elements. In this context, our study provides a fundamental reference framework that complements, rather than duplicates, previous work and supports future investigations of more compositionally complex systems.

2. Regarding the phase boundary, it is true that discrepancies exist among theoretical vs experimental results as stated by the authors (Line 39-44), and this is not surprising, as also pointed out by the authors (line 44-46). At the same time, experimental constraints about the tetragonal-cubic transition in CaSiO3 are available already (Komabayashi et al., 2007), and compositional effects have been (partially) explored too (e.g., Kurashina et al., 2004; Yu et al., 2026). Importantly, the results provided here, although these are questionable (see comment 3), appear to be in rather good agreement with one of the above-mentioned studies, i.e. Komabayashi et al. (2007). I will not comment further on this specific aspect since the authors decided to not compare their results with (Kurashina et al., 2004; Yu et al., 2026). Yet, I am afraid I see the proposed findings to be a confirmation of previous experimental studies, as also indirectly implied by the authors (line 39-42, 87-90). As such, I am afraid but I miss to see the novelty here.

We thank the reviewer for agreeing that the phase boundary of davemaoite, even for the pure $CaSiO_3$ endmember, remains controversial. As noted above, establishing the physical properties of

the endmember is essential for understanding more compositionally complex systems and therefore represents a critical step.

In this study, our goal is not to compare phase boundaries across different compositions, but rather to help resolve the discrepancy between previous XRD experiments (Komabayashi et al., 2007; Sun et al., 2016) and theoretical predictions (Shin et al., 2025; Stixrude et al., 2007; Zhang et al., 2025). Without independent approaches to address this inconsistency, it is likely to remain unresolved and continue to limit our understanding of the lower mantle.

Here, we apply an alternative experimental approach based on velocity systematics to independently constrain the phase boundary. This method provides a complementary perspective beyond existing XRD and theoretical studies, and thus represents a novel contribution in addition to the dataset itself, as noted by the reviewer. The general agreement of our results with previous XRD studies, as well as with recent theoretical calculations (Shin et al., 2025), supports the robustness of a relatively low-temperature phase transition boundary for davemaoite. Addressing and helping to resolve long-standing discrepancies constitutes an important aspect of the novelty of this study.

Detailed comments on my second point:

3. From my understanding, the phase transition boundary at P>20 GPa is determined through the interpretation of vS behavior in heating runs at HP (Line 73-81). As shown in Fib1b, vS appear to reach a maximum upon heating, followed up by a rather monotonic decrease upon further heating (Line 75-78). This vS behavior resembles that reported by (Thomson et al., 2019) at ~15 GPa and HT, and is being interpreted by the authors to be characteristic of the transition (Line 78-81). Yet, constraining the phase boundary on the interpretation of vS behavior displayed by the Brillouin scattering dataset presented here is highly problematic because:

3.1. The variation (increase/reduction) in vS magnitude is within uncertainties of individual observations, hence non resolvable, thus effectively preventing a determination of the phase boundary. For example, in their runs at ~65 and ~80 GPa , the authors assigned all observations above 700 K to the cubic phase (Fib1b; Fig 2); However, for example, the largest difference in vS magnitude is < 50 m/s between ~500 K and ~900 K, thereby within uncertainty (see also comment 6 below) of most individual observations (C4P1 and C5P1 in Ext table 1). The same applies to runs at lower pressure. This issue is partially masked by the fact that the authors decided to not report uncertainties in Figure 1b. Additionally, such apparent variations are not necessarily linked to the transition, and they could be explained in terms of:

We thank the reviewer for the careful examination of our data. The small differences of velocities at higher temperatures following the phase transition reflects the relatively weak temperature dependence. It is the relative change in velocity, rather than the absolute values alone, that

constrains the phase boundary. While individual measurements may be subject to uncertainties, the overall trend is robust and helps to minimize their influence.

3.1.1. CPO, as suggested by Extended Figure 2, in which the samples at HP/300 K display velocity variations can be up to 60 m/s along different probing directions (i.e., chi angle; line 366). Crystal preferred orientation (CPO) can influence the measured sound velocities; however, the observed variations of up to ~60 m/s suggest that the sample texture is relatively homogeneous, consistent with the XRD patterns shown in Fig. S1. This magnitude of variation is comparable to the typical uncertainties associated with sound velocity measurements, including those from ultrasonic and single-crystal Brillouin studies (Fu et al., 2023; Thomson et al., 2019). We therefore attribute these small variations primarily to natural scatter arising from minor geometric effects during sample rotation, as well as slight non-parallelism between the diamonds and the sample. In addition, during heating, measurements were consistently performed at the same position, allowing us to minimize these effects by focusing on relative changes in sound velocity.

During revision, to evaluate the effect of crystal preferred orientation on the sound velocity of davemaoite at 300 K, we synthesized davemaoite from single-crystal wollastonite using laser heating. Owing to topotactic relationships, the resulting davemaoite exhibits strong crystallographic texture. As shown in Fig. R2, the shear-wave velocity varies by more than 1.5 km/s as a function of χ angle. In comparison, the ~60 m/s variation observed in davemaoite synthesized from glass is significantly smaller, indicating a largely homogeneous texture and negligible influence of CPO. We have added additional details in Line 265-273.

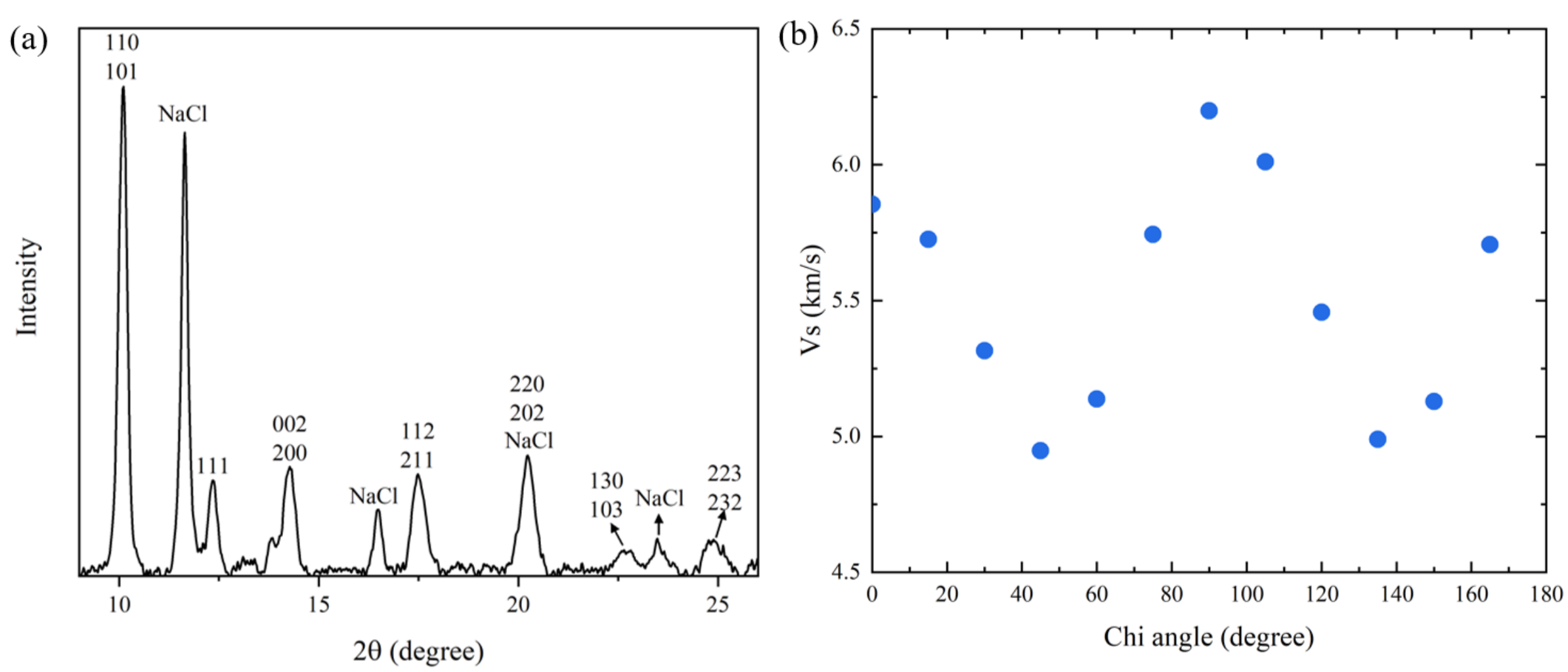

Fig. R2. (a) The XRD and (b) shear wave velocity (Vs) measurements of davemaoite synthesized from single-crystal wollastonite at 26.5 GPa 300 K. All the XRD peaks could be indexed by davemaoite and NaCl. The average Vs is ~5.52 km/s, which is consistent with davemaoite synthesized from glass (Kudo et al., 2012).

3.1.2. relatively low measurement reproducibility, as could be inferred by the data scattering as a function of pressure along a 300 K isotherm (Fig. 1a). Now, this should not come across as a surprise, since one may expect reproducibility to be as good as 0.5% of the measured velocities in single crystals and/or fluids (Sanchez-Valle et al., 2013; Speziale and Duffy, 2002), but reproducibility >0.5% on measured velocities is likely for elastically anisotropic polycrystalline material, hence the need for redundant measurements to obtain accurate results of aggregate properties (e.g., Sinogeikin et al., 2004). Unfortunately, the dataset shows that only a single observation has been collected for each P-T point at T>300K.

We fully agree that reproducibility is essential for assessing the reliability of experimental measurements. To this end, we conducted experiments using different sample assemblies with varying seat materials (ZrO_2 or Si_3N_4), which exhibit distinct thermal responses during heating. Regardless of whether pressure increased or decreased with temperature, we obtained consistent sound velocity trends, supporting the reproducibility of our results.

During the revision, we further evaluated reproducibility by loading a new cell at ~37 GPa and measuring Vs of davemaoite along both heating and cooling cycles. As temperature increased, the pressure rose gradually and overlapped with previous measurements in the ~40–45 GPa range. As shown in the updated Figs. 1–3, the data from both heating and cooling cycles are consistent with earlier results, further demonstrating the robustness and reproducibility of our measurements. We have added additional details in Line 281-284.

3.2. Generally speaking, this approach is based on a rather questionable assumption, being that the material at target has entirely transformed into a crystalline phase prior to Brillouin scattering measurements, and ultimately into the cubic polymorph with resistively heated DAC during Brillouin scattering measurements. The manuscript does not provide any direct/univoque evidence in this regard, in fact, there is a spectral contribution assigned to glass (amorphous CaSiO3, I assume) in selected spectra collected at 80 GPa (Extended Figure 3). This aspect is particularly crucial, because there is possibility that:

3.2.1. the observed peak attributed to the vS of crystalline CaSiO3 might be a superposition of the contributions from grains from both tetragonal and cubic phases especially in the proximity of a transition point, due to kinetics and/or grain interactions (e.g., Wang et al., 2023);

We fully agree that coexistence of cubic and tetragonal davemaoite is likely at temperatures near the phase boundary. However, previous ultrasonic experiments and theoretical calculations (Gréaux et al., 2019; Thomson et al., 2019; Zhang et al., 2025) consistently show that the sound velocity of davemaoite increases gradually with temperature prior to the phase transition and decreases gradually afterward. No discontinuity or abrupt velocity change is observed near the phase boundary, in contrast to the behavior reported for stishovite. Therefore, the coexistence of tetragonal and cubic davemaoite near the phase boundary is unlikely to significantly influence the

sound velocity measurements, as the two phases exhibit similar sound velocities under these conditions.

3.2.2. the phase transition might be influenced by the presence of amorphous materials (Extended Figure 3), and two-phase samples can display rather complex compressional behavior (e.g., Glazyrin et al., 2016; Trautner et al., 2026), which are not addressed in the current manuscript;

We thank the reviewer for raising the concern regarding the possible influence of residual glass on the velocity measurements. Unlike ultrasonic techniques, which determine the average sound velocity of the bulk sample and can therefore be affected by impurities or secondary phases, Brillouin spectroscopy measures the energy (frequency) shift of phonons generated by the interaction between the incident laser and the material. During this process, the incident photons either gain energy (anti-Stokes scattering) or lose energy (Stokes scattering), producing phonons with characteristic frequency shifts. The detector collects all scattered signals, including elastically scattered light without energy change (Rayleigh peak), resulting in the final Brillouin spectrum. Because different materials produce distinct frequency shifts, their corresponding peaks can be independently resolved. This is why peaks from the diamonds (anvils), pressure medium (glass), and sample (davemaoite) can all be clearly identified in our spectra. Therefore, the presence of amorphous material does not influence the measured sound velocity of the sample, as long as the peaks from different phases do not overlap, which is the case in our study (Figs. S3 and S4).

This interpretation is also supported by a recent study (Saha et al., 2026), in which bridgmanite was synthesized using laser heating. By varying heating power and duration, the authors produced both partially transformed samples containing residual glass and fully transformed samples, as confirmed by TEM analysis of quenched products. Despite these differences, the measured sound velocities obtained by Brillouin spectroscopy were consistent between the two cases.

During the revision process, we further tested the potential influence of glass on the measured velocities by systematically varying the laser-heating power and duration. As shown in Fig. S4, increasing laser power and heating duration leads to a higher intensity ratio of the sample Vs peak relative to the glass Vs peak, while the Vp peak of the glass decreases significantly, indicating progressive transformation of glass into davemaoite. Nevertheless, the position of the sample Vs peak remains unchanged, demonstrating that the presence of residual glass does not affect the measured sample velocities. In addition, the good agreement between our room-temperature results and previous studies (Gréaux et al., 2019; Kudo et al., 2012; Thomson et al., 2019) further supports the reliability of our measurements.

4. Regarding the implications, the current paper aims at estimating the effect that volumetric abundance of $CaSiO_3$ has on the vS of lower mantle isotropic aggregates at 40 and 100 GPa (and I assume 300 K, since I was not able to find an explicit mention in the manuscript). For each pressure at target, the authors assumed a given composition for bridgmanite (bdm) and ferropericlase (fp). For simplicity (Line 155), the authors also assumed the electronic configuration of Fe^{2+} in fp to be high spin and low spin, at 40 and 100 GPa, respectively. In short,

the authors point out that a CaSiO3-bearing aggregate would display lower vS than a CaSiO3 - free aggregate. This is particularly evident at relatively high pressures, prompting the authors to suggest that a normal lower mantle is likely depleted in CaSiO3. Furthermore, the authors suggest that CaSiO3 davemaoite may be concentrated in the LLSVPs, a scenario that could be explained through the crystallization of residual Ca-rich melts segregated in the deepest regions of the mantle. I am afraid that the evidence supporting these claims are difficult to validate and replicate, mostly due to relatively simplistic approach the authors followed and scarcity of details presented, making the implication section feel highly speculative. For example:

4.1. The authors model Al-bearing bdm. It is unclear if the authors modeled bdm as a multi-component system, and if so, there is no information reported regarding the molar fractions of FeAlO3, FeFeO3 and AlAlO3 components which one may expect to be employed in such modelling. The information reported in extended table 3 is not sufficient to validate their results;

We thank the reviewer for pointing out the confusion regarding our treatment of the compositional dependence of bridgmanite elastic properties. Because the definition of distinct endmembers is not unique and the elastic properties of pure endmembers remain incompletely constrained, we did not calculate the elastic properties of bridgmanite using endmember mixing models. Instead, we directly adopted parameterizations from previous studies (Fu et al., 2023; Murakami et al., 2024), which describe the variation of elastic properties as a function of Fe and Al contents. We have clarified this point in Line 172-174.

4.2. Calculations are performed at HP/300 K despite the authors having thermoelastic parameters. As such, temperature effects are not considered, despite these being critical (e.g., Deng et al., 2023).

As discussed in the "Elastic properties of cubic davemaoite" section and shown in Fig. 3, we calculated the sound velocities along a mantle adiabat geotherm (Katsura, 2022). We have further emphasized this in Line 175 and Fig. 4.

4.3. There is mounting experimental and seismological evidence that fp displays a mixed-spin state across the entire lower mantle and spin fraction varies with depth (e.g., Cobden et al., 2024; Méndez et al., 2022; Trautner et al., 2024, 2023). This effect is not captured by the modelling approach proposed by the authors thereby lowering the overall accuracy of their results;

We fully agree that the spin transition in ferropericlase is complex and can strongly influence its elastic behavior. However, during the spin transition, the most significant effect is a reduction in the bulk modulus over a broad pressure range, which primarily affects Vp (Cobden et al., 2024; Trautner et al., 2023). In contrast, the spin transition mainly modifies the pressure dependence of the shear modulus without producing abrupt changes or significant softening (Fu et al., 2023; Wu et al., 2013). Furthermore, the influence of mixed-spin states on Vs is restricted to a relatively narrow pressure interval (~10 GPa, Wu et al. 2013). Because our model primarily relies on Vs, the

complex behavior associated with mixed-spin states in ferropericlase is unlikely to significantly affect our results. We have clarified this point in Line 165-168.

4.4. The origin and nature of LLSVPs is still highly debated, despite authors' claims (Line 99-101)- on the top of my head for example it has been recently suggested that they might have a thermal origin (Muir et al., 2026);

We thank the reviewer for highlighting the complexity of the origin of LLSVPs. Given the range of proposed mechanisms, LLSVPs may involve multiple contributing processes, and enrichment in davemaoite could represent one possible factor. The primary focus of this study is to constrain the properties and implications of davemaoite in the ambient lower mantle, rather than to propose a unique explanation for the origin of LLSVPs. We have added a paragraph in Line 204-206 to clarify this point.

4.5. The proposed dataset does not provide information on elastic anisotropy, hence the authors are forced to model isotropic aggregates. Therefore, dynamics cannot be considered and/or discussed, yet these are crucial to understand lower(most) mantle seismic signature & mineralogy (e.g., Gay et al., 2024), including LLSVPs (e.g., Deng et al., 2017).

We agree that seismic anisotropy is important in regions characterized by strong mantle flow, such as subduction zones and the core–mantle boundary. However, the focus of this study is the ambient lower mantle, where mantle flow is relatively weak and seismic properties are generally considered to be more isotropic. Even in previous single-crystal studies, including single-crystal Brillouin measurements and theoretical calculations (Criniti et al., 2021; Fu et al., 2023; Zhang et al., 2025), modeled seismic velocities are commonly derived from isotropic aggregate elastic properties.

We agree that future investigations using single-crystal davemaoite could provide additional insights into elastic anisotropy. However, given the experimental challenges associated with synthesizing high-quality single crystals of davemaoite, such efforts remain technically demanding and are beyond the scope of the present study.

I would like to add a general comment, as well as major and minor corrections. I hope the authors would consider them independently on the venue in which the manuscript will be ultimately published.

We thank the reviewer for the suggestions and have tried to improve the manuscript accordingly.

General comments:

5. I found several arguments the authors make to be hard to follow and I would like to suggest the authors to improve readability of their manuscript through rephrasing. As an example, Line 27-30, the authors state that "because of direct sampling of the lower mantle is not feasible, our understanding relies primarily on seismic observations, which in turn require robust constraints on the elastic properties of relevant minerals under high-pressure high-temperature conditions".

Such statement may be unclear to the reader for the following reasons: a) it is unclear why sampling of the lower mantle is not feasible (I would suggest the authors to either clarify this aspect to the reader, or simply omit this part); b) it is unclear what information can be extracted by seismic observations (e.g., current thermo-physical state of the mantle? Mineralogical, chemical composition of the mantle?); c) the contribution that "robust" constraints on physical properties of mantle minerals may provide to this topic is also unclear (why would these constraints help with observations? I mean, observations remain observations, it's all about their interpretation…). Furthermore, the authors may want to employ a more appropriate language to describe their research, for example: a) I do not necessarily believe that the term robust is appropriate here – the authors could say accurate and precise constraints instead); b) the use of "relevant minerals" cut out all those materials that are not mineral (e.g., phases), yet are present in the lower mantle and may contribute to the seismic signature of the lower(most) mantle. Similarly, I found hard to follow and I suggest rephrasing in:

We thank the reviewer for pointing out the unclear expression and have revised the sentence accordingly in Line 27-30.

5.1. Line 31-38, I would suggest the authors to not employ the term progressively since the kinetics of this transition are not well explored in literature and/or in the present study. Also, I think it would be appropriate to say that it would be important to define the boundary of this transition in a P-T space relevant to the lower mantle (line 36-38), which in the end is what the authors do in their research.

We modified the sentence accordingly in Line 33 and 37.

5.2. Line 39-49. I think this subsection could benefit from rephrasing, because it starts by saying that ''the phase boundary is poorly constrained", but then argue that experimental and theoretical results are available. The authors could, for example, remove the statement in line 39, and simply discuss discrepancies between experimental and theoretical studies. Furthermore, I would suggest the authors to clarify and expand their statement regarding "independent experimental constraints" because I find it unclear. Ultimately, I would like to note that the character of this transition in CaSiO3 has yet to be explored, but it may be worth having a look and include in the discussion spontaneous strains studies coupled to Landau theory on tetragonal-cubic transition in perovskites (Carpenter and Salje, 1998; Perks et al., 2014), and of course keep in mind the elastic softening reported in CaSiO3 (Zhang et al., 2025).

We thank the reviewer for the helpful suggestions for clarification and have revised the sentence accordingly in Line 39-40. To better describe the nature of this transition, we also added further comparison with the $CaTiO_3$ system in line 80-82. The behavior of the $CaSiO_3$ system differs substantially from that of $CaTiO_3$, as demonstrated by previous ultrasonic studies (Thomson et al., 2019). In particular, Thomson et al. (2019) investigated both $CaTiO_3$ and $CaS_{0.6}Ti_{0.4}O_3$ and showed that Ti-bearing davemaoite exhibits significant elastic softening, whereas pure $CaSiO_3$ does not display comparable softening behavior. Our results are fully consistent with previous ultrasonic

measurements (Gréaux et al., 2019; Thomson et al., 2019). Zhang et al. (2025) also acknowledge that the origin of the discrepancy between their calculations and the experimental observations remains unresolved, stating that "the exact cause of this discrepancy is currently unknown." Therefore, future theoretical investigations with improved constraints may help clarify the source of these differences. In addition, similar discrepancies have also been reported for the elastic softening of stishovite (Krymarys et al., 2025; Yang and Wu, 2014), which may suggest broader limitations in current theoretical approaches under these conditions.

Specifically, regarding the theoretical calculations of Zhang et al. (2025), we would like to offer several comments. Zhang et al. (2025) argue that their room-temperature results are reliable because they are consistent with previous experimental measurements. However, their predicted high-temperature velocities differ substantially from the available experimental data, which they attribute to anisotropy in cubic davemaoite.

We find this interpretation difficult to reconcile with previous studies. Theoretical calculations consistently indicate that tetragonal davemaoite is also strongly anisotropic (Stixrude et al., 2007; Tsuchiya, 2011; Zhang et al., 2025), as further illustrated by our results in Fig. R2. If agreement between calculations and experiments at room temperature is taken as evidence for reliable isotropic behavior, it is not immediately clear why discrepancies at high temperature should instead be attributed primarily to CPO in the measured cubic davemaoite.

In particular, in multi-anvil experiments, the high-temperature measurements are typically conducted immediately after synthesis, as the davemaoite phase forms at elevated temperatures. It is therefore difficult to understand why the initially synthesized high-temperature samples would exhibit significant CPO effects, whereas the same samples would later display nearly isotropic behavior during subsequent room-temperature measurements.

Although theoretical calculations are outside our primary area of expertise, we would nevertheless like to note these concerns regarding the interpretation and applicability of the results presented in Zhang et al. (2025).

Major corrections:

6. I am concerned that vS uncertainties have been underestimated since most, if not all, uncertainties reported are below 1% (Extended Table 1), including in spectra of relatively poor quality (e.g., 50 m/s uncertainty at 81 GPa, 973 K; spectrum in extended figure 3). I would like to note that Brillouin spectra of polycrystalline sample result from a superposition of light scattered from all grains contained within a scattering volume (e.g., Angel et al., 2009; Sinogeikin et al., 2004; Speziale et al., 2014). Due to the relatively large elastic anisotropy of the cubic polymorph, with a vS anisotropy that can be as large as 31% (Zhang et al., 2025), as well as the anisotropy of the photo-elastic coupling intrinsic to Brillouin scattering measurements, one may expect that the observed spectral features attributed to the vS of CaSiO3 would be associated to peaks having a

relatively broad, potentially asymmetric shape, especially when collected in a relatively short time as done in this study (10-30 min, line 374). This seems to be indeed the case, as shown in the spectra presented in Extended Figure 3. All of these aspects should be considered in the estimation of uncertainties; as such, it would be sensible to propagate both statistical uncertainties resulting from the peak fitting, take into account the standard deviation between stokes and anti-stokes spectral contributions, and ultimately propagate the systematic error induced by the scattering angle calibration as well (0.1 deg; line 354). To validate their results, I would like to suggest the authors to disclose the approach followed to: a) carry out the channel-to-velocity conversion; b) uncertainty determination in observed velocities; if scanning amplitude determination was done through FP1 peak fitting in tandem, I would suggest the authors to report at least one spectrum, collected in tandem, in which the quality of FP1 and FP2 spectral contributions can be evaluated.

We thank the reviewer for raising the concern regarding the uncertainties in the Brillouin measurements. No smoothing or filtering was applied to the spectra presented in the manuscript, which may make them appear noisier than those shown in some previous studies. Nevertheless, the peak fitting remains well constrained. For the sharp peaks observed at lower temperatures, the fitting uncertainty is typically ~0.02–0.03 GHz, corresponding to ~10–20 m/s in sound velocity. At higher temperatures (e.g., 973 K), the broader signals yield fitting uncertainties of ~0.05–0.06 GHz, corresponding to ~30–40 m/s.

Crystal anisotropy itself does not lead to significant broadening of the Brillouin peaks. As shown in Fig. R2 and predicted by theoretical calculations (Stixrude et al., 2007; Zhang et al., 2025), tetragonal davemaoite exhibits substantial elastic anisotropy. However, the room-temperature Brillouin peaks shown in Fig. S3 remain sharp, indicating that anisotropy does not significantly contribute to peak broadening or additional fitting uncertainty. Furthermore, because all high-temperature measurements were collected at the same sample position and geometry, neither angle calibration nor velocity variations with χ angle should affect the observed temperature-dependent velocity trends.

Therefore, the velocity uncertainties reported in the table primarily reflect the fitting uncertainty of the Brillouin peaks and the small differences between the Stokes and anti-Stokes signals. Even considering the angle-calibration uncertainty of 0.1°, the resulting velocity uncertainty would only be ~10 m/s and would not significantly affect the conclusions. Following the reviewer's suggestion, we have added a velocity axis to Fig. S3 and included additional discussion in Text S2 to clarify the uncertainty estimation for the velocity measurements.

Regarding the influence of FP1 and FP2, we may have misunderstood the reviewer's concern and would like to clarify the experimental procedure. The scan amplitude is determined using FP1 under alignment mode. In tandem mode, where the actual Brillouin spectra are collected, signals are observed only when FP1 and FP2 are properly merged; otherwise, no Brillouin signal can be detected if the two peaks are separated. If the reviewer is instead referring to the calibration spectra obtained in alignment mode, where FP1 and FP2 can be individually resolved, we have added Fig.

S5 to demonstrate the complete separation of FP1 and FP2 during the determination of the scan amplitude.

7. It is unclear if laser-heating, hence crystallization of CaSiO3, was achieved once the DAC was already pre-compressed to the target pressure. The only piece of information I found is that each DAC was pre-compressed to at least 35 GPa (Line 345, Extended Figure 1). Since texturing is induced through compression (Wenk et al., 2004), and cubic CaSiO3 is relatively weak (Immoor et al., 2022), I think it would be important to provide further supporting evidence that no texturing was induced in CaSiO3 prior to Brillouin measurements, that is, DACs were not further compressed after CaSiO3 synthesis, especially NaCl-free DACs. At the moment, the only evidence provided regards 300K measurements on DACs pre-compressed at 29 and 74.5 GPa (Extended figure 2), and the latter was not used for HT measurements I assume (C1P1 is the only cell at 29 GPa in Extended table 1).

I think it is worth checking that none of the DACs contained textured samples before HT runs, since each sample may be different in term of texture and grain size. Therefore, I suggest the authors replicate what done for C2P1, that is, I suggest the authors to provide an angular dispersion curve of vS at 300 K for each one of the DAC that was later employed for HT runs.

We thank the reviewer for pointing out the possibility of texture development during compression. For each cell, laser heating was performed after compression to the target pressure. Since all cells except C2 were used for only a single pressure condition, any texture development during compression has no influence on the results. Even for C2, which was measured at two pressure points, the sample was re-heated after further compression to resynthesize davemaoite. The resulting velocity variations with χ angle remained small. We have added additional clarification in Line 237-242.

During the revision, following the reviewer's suggestion, we further added more χ-angle measurements for different cells. As shown in Fig. S2, the velocity variations are consistently within ~60 m/s. Unfortunately, due to the limited size of the sample chamber, it was not possible to obtain reliable signals for all χ angles at the highest-pressure condition. Nevertheless, the consistently small variations observed in the other cells demonstrate that our synthesis approach produces samples with relatively homogeneous textures. This interpretation is further supported by the uniform XRD patterns and the good agreement between our measured sound velocities and those reported in previous studies.

Minor corrections

8. Figure 1a: I suggest the authors to report their fit of velocity data in Figure 1a, not just their

observations; This would give the reader a direct mean to visualize potential discrepancies with previous experimental studies as currently discussed in Line 120-123.

We did not directly compare the 300 K fitting results because the fitting equations used in this study differ slightly from those adopted in previous room-temperature studies (Gréaux et al., 2019; Kudo et al., 2012). During the revision, we added fits to our data in Fig. 1 using the same equations as those employed in the previous studies to facilitate a more direct comparison.

9. I suggest the authors to clarify the correction applied to high-P/T velocity data to validate their results; I further suggest the authors to report both non-corrected and corrected values as supplementary material.

The velocity corrections shown in Fig. 1b for 300 K tetragonal davemaoite and high-temperature cubic davemaoite are based on the fitted pressure dependences obtained in this study. Because the pressure dependences of the tetragonal and cubic phases differ slightly, the velocity corrections for temperatures above 300 K but below the phase boundary were estimated by assuming a linear variation in pressure dependence with temperature. We have added this information to Text S3.

For the detailed values shown in each plot of the main text, we have uploaded a separate data file containing the relevant information, including the corrected values used in Fig. 1b.

10. Line 77-81, in fact, one may expect a decrease of G in the proximity of the transition (Zhang et al., 2025) , which should be reflected in a local minimum of vS at the transition point. Elastic softening may also be present in the cubic phase (Zhang et al., 2025);

As we mentioned in point 5.2, we have added more discussion for the softening behaviors of davemaoite.

11. Line 82-83, this is obvious since the phase boundary was determined in combination with results from ultrasonic measurements as well; I suggest the authors to remove this statement.

We have revised the sentence accordingly in Line 85.

12. Line 94-96, I suggest the authors to further clarify that this is a confirmation of previous XRD, as previously stated in line 39-42;

We have revised the sentence accordingly in Line 98.

13. Line 97-101, the nature of LLSVP and ULVZ is highly debated (McNamara, 2019), and more recently (Muir et al., 2026). I suggest the authors to improve the current discussion on the state-of-the-art regarding LLSVP and ULVZ so to place the manuscript in a more updated context.

We thank the reviewer for highlighting the complexity of LLSVPs and have added a paragraph in Line 204-206 to further discuss this point.

14. Line 101-108, I do not agree with this statement. From my understanding, the phase boundary by (Zhang et al., 2025) does not intersect the normal mantle geotherm at the base of the lower mantle, in fact, it is even below the cold slab geotherm, see their Figure 2. I suggest them to clarify their statement.

We thank the reviewer for pointing out the confusion. We believe there may be some inconsistency in the definition of the phase boundary in Zhang et al. (2025). Previously, we interpreted the upper bound shown in Fig. 2—where cubic davemaoite begins to exhibit normal elastic behavior—as the effective phase boundary, allowing it to intersect the mantle geotherm.

Alternatively, if the lower bound is interpreted as the actual phase boundary and the elastic softening region is assumed to occur entirely within the cubic phase field, then the experimentally constrained transition boundary in this study (533 K + 2.4 K/GPa) would correspond to a transition temperature of only ~860 K at the core–mantle boundary. Even when considering an additional softening interval of 500–800 K, the resulting temperature range would reach at most ~1700 K, which remains well below the coldest slab geotherm (~1900 K). This suggests that such elastic softening, if present, would still be unlikely to occur under lower-mantle conditions. We have added further clarification in Line 104-111.

15. Line 368-369 I do not necessarily agree that cubic CaSiO3 is nearly elastically isotropic, since it contrasts with recent literature (Zhang et al., 2025). I suggest the expand this aspect through discussion.

We thank the reviewer for pointing this out. Our previous statement regarding isotropic properties was based on earlier first-principles calculations by Stixrude et al. (2007), which predict anisotropic behavior that differs from the results of Zhang et al. (2025). Since we are unable to assess which study provides the more accurate description, we have removed the statement. Future studies may help clarify this issue.

References

Cobden, L., Zhuang, J., Lei, W., Wentzcovitch, R., Trampert, J., Tromp, J., 2024. Full-waveform tomography reveals iron spin crossover in Earth's lower mantle. Nat Commun 15, 1961. https://doi.org/10.1038/s41467-024-46040-1

Criniti, G., Kurnosov, A., Boffa Ballaran, T., Frost, D.J., 2021. Single-Crystal Elasticity of MgSiO3 Bridgmanite to Mid-Lower Mantle Pressure. Journal of Geophysical Research: Solid Earth 126, e2020JB020967. https://doi.org/10.1029/2020JB020967

Fu, S., Zhang, Y., Okuchi, T., Lin, J.-F., 2023. Single-crystal elasticity of (Al,Fe)-bearing bridgmanite up to 82 GPa. American Mineralogist 108, 719–730. https://doi.org/10.2138/am-2022-8435

Gréaux, S., Irifune, T., Higo, Y., Tange, Y., Arimoto, T., Liu, Z., Yamada, A., 2019. Sound velocity of CaSiO3 perovskite suggests the presence of basaltic crust in the Earth's lower mantle. Nature 565, 218–221. https://doi.org/10.1038/s41586-018-0816-5

Katsura, T., 2022. A Revised Adiabatic Temperature Profile for the Mantle. Journal of Geophysical Research: Solid Earth 127, e2021JB023562. https://doi.org/10.1029/2021JB023562

Komabayashi, T., Hirose, K., Sata, N., Ohishi, Y., Dubrovinsky, L.S., 2007. Phase transition in CaSiO3 perovskite. Earth and Planetary Science Letters 260, 564–569. https://doi.org/10.1016/j.epsl.2007.06.015

Krymarys, E., Murakami, M., Saha, P., Liebske, C., 2025. The presence of ancient subducted oceanic crust contributes to seismic anomalies in Large Low Shear Velocity Provinces. Commun Earth Environ 6, 713. https://doi.org/10.1038/s43247-025-02700-8

Kudo, Y., Hirose, K., Murakami, M., Asahara, Y., Ozawa, H., Ohishi, Y., Hirao, N., 2012. Sound velocity measurements of CaSiO3 perovskite to 133 GPa and implications for lowermost mantle seismic anomalies. Earth and Planetary Science Letters 349–350, 1–7. https://doi.org/10.1016/j.epsl.2012.06.040

Murakami, M., Khan, A., Sossi, P.A., Ballmer, M.D., Saha, P., 2024. The Composition of Earth's Lower Mantle. Annual Review of Earth and Planetary Sciences 52, 605–638. https://doi.org/10.1146/annurev-earth-031621-075657

Ono, S., Mibe, K., Ohishi, Y., 2014. Raman spectra of culet face of diamond anvils and application as optical pressure sensor to high temperatures. Journal of Applied Physics 116, 053517. https://doi.org/10.1063/1.4891681

Saha, P., Murakami, M., Miyajima, N., 2026. Earth's Lower Mantle Predominated by Ferric Iron-Rich Bridgmanite Inferred From High Pressure Elasticity Measurements. Geophysical Research Letters 53, e2025GL118109. https://doi.org/10.1029/2025GL118109

Shin, Y., Di Lucente, E., Marzari, N., Monacelli, L., 2025. The thermodynamics of CaSiO3 perovskite in Earth's lower mantle. Phys. Rev. B 112, 174113. https://doi.org/10.1103/3vgz-fnth

Stixrude, L., Lithgow-Bertelloni, C., Kiefer, B., Fumagalli, P., 2007. Phase stability and shear softening in CaSiO3 perovskite at high pressure. Phys. Rev. B 75, 024108. https://doi.org/10.1103/PhysRevB.75.024108

Sun, N., Mao, Z., Yan, S., Wu, X., Prakapenka, V.B., Lin, J.-F., 2016. Confirming a pyrolitic lower mantle using self-consistent pressure scales and new constraints on CaSiO3 perovskite. Journal of Geophysical Research: Solid Earth 121, 4876–4894. https://doi.org/10.1002/2016JB013062

Thomson, A.R., Crichton, W.A., Brodholt, J.P., Wood, I.G., Siersch, N.C., Muir, J.M.R., Dobson, D.P., Hunt, S.A., 2019. Seismic velocities of CaSiO3 perovskite can explain LLSVPs in Earth's lower mantle. Nature 572, 643–647. https://doi.org/10.1038/s41586-019-1483-x

Trautner, V.E., Stackhouse, S., Turner, A.R., Koelemeijer, P., Davies, D.R., Méndez, A.S.J., Satta, N., Kurnosov, A., Liermann, H.-P., Marquardt, H., 2023. Compressibility of ferropericlase at high-temperature: Evidence for the iron spin crossover in seismic tomography. Earth and Planetary Science Letters 618, 118296. https://doi.org/10.1016/j.epsl.2023.118296

Tsuchiya, T., 2011. Elasticity of subducted basaltic crust at the lower mantle pressures: Insights on the nature of deep mantle heterogeneity. Physics of the Earth and Planetary Interiors, Proceedings of the 12th Symposium of SEDI 188, 142–149. https://doi.org/10.1016/j.pepi.2011.06.018

Wu, Z., Justo, J.F., Wentzcovitch, R.M., 2013. Elastic Anomalies in a Spin-Crossover System: Ferropericlase at Lower Mantle Conditions. Phys. Rev. Lett. 110, 228501. https://doi.org/10.1103/PhysRevLett.110.228501

Yang, R., Wu, Z., 2014. Elastic properties of stishovite and the CaCl2-type silica at the mantle temperature and pressure: An ab initio investigation. Earth and Planetary Science Letters 404, 14–21. https://doi.org/10.1016/j.epsl.2014.07.020

Zhang, C., Yang, J.-Y., Sun, T., Zhang, H., Brodholt, J.P., 2025. Strong precursor softening in cubic CaSiO3 perovskite. Proceedings of the National Academy of Sciences 122, e2410910122. https://doi.org/10.1073/pnas.2410910122

Zhou, W.-Y., Hao, M., Su, W., Kim, T., Chen, S., Shim, S.-H., Zhang, D., Nguyen, P.Q.H., Armstrong, K., Zhang, J.S., 2025. Elasticity of davemaoite as a primary contributor to lower-mantle heterogeneities. Science 390, 935–939. https://doi.org/10.1126/science.adx8356

Response to the Reviewers

We thank the fourth reviewer for the comments. The detailed response is listed below (his/her points are in blue; our answers are in black). All revisions made to the manuscript and Supplementary Materials are highlighted in blue, and revised figures are indicated by blue titles.

Response to comments by Reviewer #4:

Reviewer #4 (Remarks to the Author):

The manuscript by Hao et al. presents new high-pressure and high-temperature Brillouin spectroscopy measurements of $CaSiO_3$ davemaoite up to 80 GPa and 973 K. By combining these new elasticity measurements with previously published volume data, the authors derive an updated thermoelastic model for cubic davemaoite and discuss its implications for lower-mantle mineralogy and the origin of Large Low Shear Velocity Provinces (LLSVPs).
The manuscript addresses an important problem in mineral physics and deep-Earth geophysics. Reliable sound velocity measurements under true lower-mantle pressure–temperature conditions are essential not only for constraining the elastic properties and mineralogy of the Earth's lower mantle, but also because they provide one of the few experimental probes capable of detecting structural phase transitions that are difficult to resolve using conventional X-ray diffraction. This is particularly relevant for the tetragonal-to-cubic transition in $CaSiO_3$ davemaoite, where the structural distortion is subtle and diffraction peak splitting becomes increasingly difficult to resolve at high pressures and temperatures. In this respect, high-pressure Brillouin spectroscopy represents a powerful complementary technique, and the present study has the potential to provide an important contribution to our understanding of both the phase transition and the thermoelastic properties of davemaoite under lower-mantle conditions.
Overall, I find the work technically impressive, well executed, and potentially suitable for publication in Nature Communications. However, several important issues should be addressed before the conclusions can be fully supported. In particular, the interpretation of the observed temperature dependence of the shear-wave velocity as direct evidence for the tetragonal-to-cubic phase transition requires additional justification. I therefore recommend major revision.

We thank reviewer #4 for the supportive comments and suggestions. During the revision, we have added more information to clarify the experimental results.

Major Comment
A major strength of this study is the use of high-pressure high-tempearature Brillouin spectroscopy as an alternative probe for detecting the tetragonal-to-cubic phase transition in davemaoite, which is difficult to resolve by X-ray diffraction under lower-mantle conditions. Because this interpretation forms the basis of several key conclusions of the manuscript, it is essential to demonstrate convincingly that the observed temperature dependence of the shear-wave velocity is uniquely associated with the phase transition and cannot arise from

experimental artefacts.
The authors interpret the observed increase of the shear-wave velocity during the initial stage of heating, followed by a decrease above a characteristic temperature, as the transition from tetragonal to cubic davemaoite. While this interpretation is plausible and is consistent with previous ultrasonic studies, an increase of shear-wave velocity with increasing temperature is nevertheless unusual for perovskite-type materials. Therefore, alternative explanations should be carefully excluded.
During the experiments, pressure was determined from the Raman edge of the diamond anvils, whereas temperature was monitored by thermocouples attached to the gasket. Both pressure and temperature influence the Raman signal of diamond, and pressure variations associated with thermal expansion of the diamond anvil cell during resistive heating are well known. I appreciate that the authors performed experiments using anvils mounted on seats with different thermal expansion coefficients and provide a careful discussion of pressure uncertainties in the Supplementary Information. Nevertheless, I believe that additional experimental evidence would considerably strengthen the manuscript.
In particular, I encourage the authors to demonstrate that the anomalous temperature dependence is unique to davemaoite. Whenever possible, Brillouin peaks originating from the residual glass and/or the pressure-transmitting medium should be presented and compared with the behaviour of the davemaoite peak under identical experimental conditions. If these peaks exhibit the expected monotonic decrease in velocity with increasing temperature while only davemaoite displays the initial increase in velocity, this would provide compelling evidence that the observed behaviour is intrinsic to the sample rather than an artefact related to pressure determination or pressure-temperature correlations in the Raman calibration.

We thank the reviewer for highlighting the importance of demonstrating that the anomalous sound-velocity behavior observed in davemaoite at high temperature is not an experimental artifact. To address this concern, we have added Fig. S6 (shown here as Fig. R1), which presents representative shear-wave velocity (Vs) measurements of the $CaSiO_3$ glass collected simultaneously with the sample during the high-temperature experiments. The figure shows both the raw Vs data obtained during the heating–cooling cycle, during which pressure varied slightly with temperature, and the values corrected to a fixed pressure using the experimentally determined pressure dependence of Vs for the same glass under room-temperature conditions (Geballe et al., 2022).

Although the temperature dependence of sound velocity in glass can be complex and may be influenced by factors such as thermal history and synthesis conditions, the measured Vs varies monotonically with temperature throughout the experiment. This behavior contrasts with the anomalous velocity change observed in davemaoite, supporting our interpretation that the latter reflects an intrinsic phase transition rather than an experimental artifact.

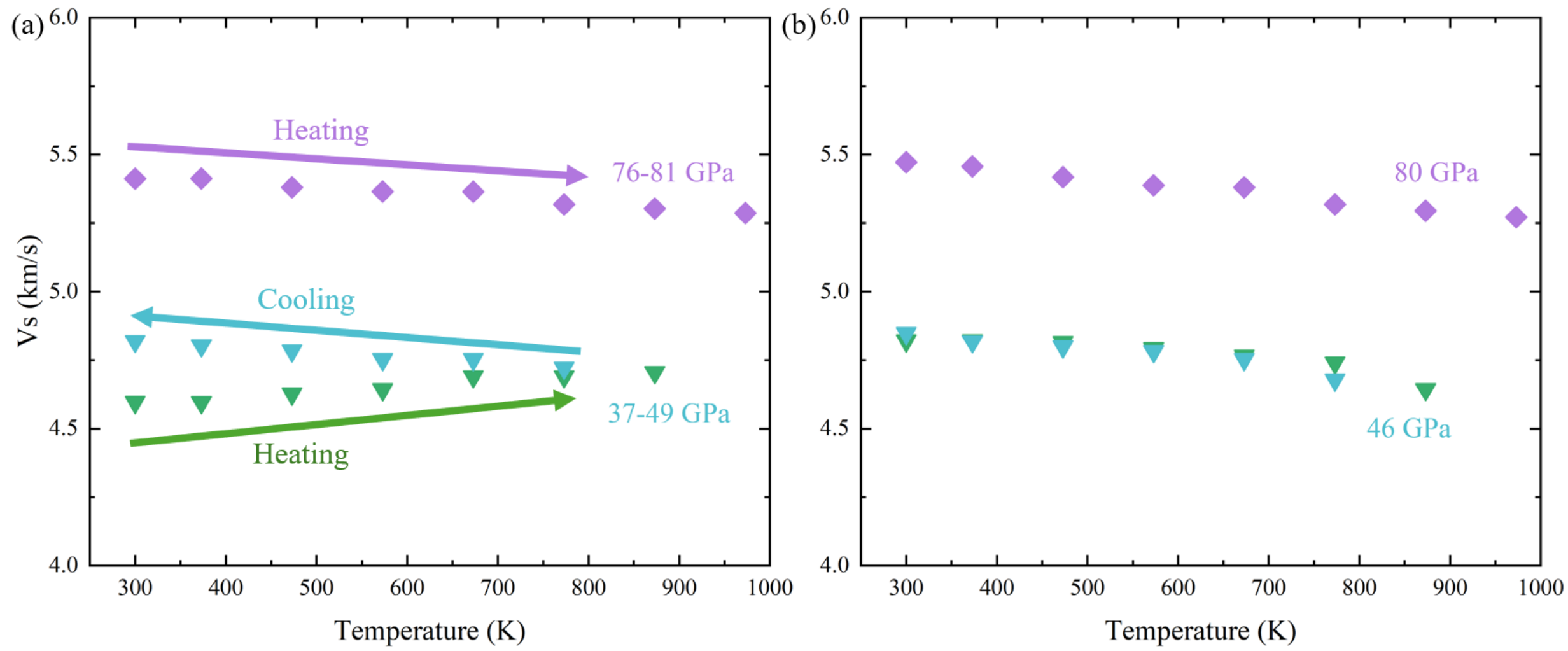

Fig. R1. Representative Vs measurements of the glass collected together with the sample under high-temperature conditions. (a) Measured Vs of the glass during heating and cooling, without correction for pressure variations induced by temperature changes. (b) Vs corrected using the room-temperature pressure dependence from previous experiments (Geballe et al., 2022).

In addition, the manuscript would benefit from a more comprehensive discussion of the possible physical origin of the positive temperature dependence of the shear-wave velocity in tetragonal davemaoite. The manuscript provides only a brief discussion of the underlying mechanism. Expanding this discussion and placing the observations into the broader context of previous experimental and theoretical studies would strengthen the interpretation considerably.

We thank the reviewer for the suggestion to further explain the physical mechanisms underlying the anomalous elastic behavior of davemaoite. Early theoretical calculations (Stixrude et al., 2007) suggested that the asymmetric response of tetragonal davemaoite to strain may induce additional octahedral rotations, resulting in elastic softening. Subsequent ultrasonic studies on $BaTiO_3$, $(Ca,Sr)TiO_3$, and $Ca(Ti,Si)O_3$ proposed that the elastic softening of tetragonal davemaoite may instead be related to a temperature-activated twin-domain-wall process (Perks et al., 2014; Salje, 2013; Thomson et al., 2019).

Regardless of which mechanism better explains the elastic differences between the cubic and tetragonal phases, X-ray diffraction measurements demonstrate that tetragonal davemaoite progressively approaches cubic symmetry with increasing temperature below the phase boundary (Komabayashi et al., 2007). This gradual increase in structural symmetry is accompanied by a continuous increase in sound velocity prior to the phase transition. The excellent agreement between the present Brillouin spectroscopy measurements and previous ultrasonic studies (Gréaux et al., 2019; Thomson et al., 2019) further supports the conclusion that the positive temperature dependence of sound velocity in tetragonal davemaoite is a real physical phenomenon rather than an experimental artifact. We have added this discussion to Lines 85–90 of the revised manuscript.

Minor Comments

1. Line 51: The phrase "absolute elastic properties" is unclear. Do the authors refer to discrepancies between experiments and theoretical predictions?

Yes, we have improved the sentence in Line 51.

2. Lines 77–79: The wording "progressive transformation" is thermodynamically ambiguous. Strictly speaking, a phase transition itself is not progressive. If the transition extends over a finite temperature interval, this should reflect phase coexistence resulting from kinetic effects, pressure gradients, or temperature gradients within the sample. I suggest rephrasing this section accordingly.

We thank the reviewer for the clarification. We have revised the section accordingly.

3. Line 79: The sentence "Once temperatures exceed the phase boundary..." assumes the interpretation before presenting the observations. It would be more objective first to describe the experimental observation (e.g., "Above a certain temperature, the measured shear-wave velocity decreases monotonically...") and only then discuss its interpretation in terms of the phase transition.

We have revised the text accordingly in Lines 79 and 83–85 of the revised manuscript.

4. Line 247: "...davemaoite is supposed to by tetragonal..." → "...supposed to be tetragonal..."

We have revised the typo in Line 253.

5. Methods: The observation that the thermocouple reading increases by 1–2 °C demonstrates the sensitivity of the thermocouple but does not necessarily reflect the actual sample temperature. Did the authors monitor the thermocouple response during CO_2-laser heating used for sample synthesis? Conversely, the observed sensitivity may also indicate a certain temperature increase induced by the probing laser itself. Although this would likely not affect the conclusions because identical laser power was used throughout the Brillouin measurements, it may be worth mentioning as a consideration for future experiments.

We thank the reviewer for raising the issue of potential laser heating. We did not measure the sample temperature using thermocouples during the CO_2 laser-heating experiments. The laser-heated spot was relatively small (~10 μm), considerably smaller than the sample chamber thickness, making it difficult for thermocouple measurements to accurately reflect the actual temperature within the heated region.

Unlike the CO_2 laser, which is absorbed by $CaSiO_3$ and serves as the heating source, the green laser used for Brillouin spectroscopy is expected to be largely transparent to the sample. Consequently, heating induced by the Brillouin laser should be minimal. This interpretation is supported by the excellent agreement between our room-temperature sound velocity measurements and previous studies (Gréaux et al., 2019; Kudo et al., 2012), suggesting that any

laser-heating effect associated with the Brillouin probe laser is negligible under our experimental conditions. Nevertheless, we agree that systematic measurements of sound velocity as a function of Brillouin laser power would be valuable for further evaluating the potential influence of laser heating. We have revised the manuscript accordingly in Lines 264–267.

6. Supplementary Fig. S5: I could not find any reference to this figure in either the main text or the Supplementary Information, and its purpose is not clear. The figure appears to show a standard Fabry–Pérot calibration spectrum acquired in alignment mode, where interaction between FP1 and FP2 is not expected. Rather than including this figure, I suggest that the authors provide a brief description of the scattering-angle calibration procedure. The Supplementary Information states that an angular uncertainty of 0.1° contributes only ~10 m/s to the velocity uncertainty, but the basis for this estimate is not explained. A short description of the calibration procedure would improve the transparency and reproducibility of the experimental methodology. Since this uncertainty is systematic, it does not affect the observed temperature-dependent trends or the qualitative conclusions of the study; however, it contributes to the uncertainty of the absolute sound velocities and, consequently, to the derived thermoelastic parameters.

We agree that it is important to provide additional details regarding the scattering-angle calibration procedure. Accordingly, we have replaced the original Fig. S5 with representative Brillouin spectra of the borosilicate crown optical glass (BK7) used for angle calibration. To accurately determine the scattering angle, a 50 µm-thick, double-side-polished BK7 glass wafer with a well-characterized sound velocity (Vp = 5965 m/s) from high-precision ultrasonic measurements was positioned at the sample location and measured under identical experimental conditions. The measured Brillouin frequency shifts were then used to determine the actual scattering angle following the procedure described by Murakami et al. (2009). The uncertainty of the calibrated angle was estimated from the uncertainty in fitting the Brillouin peaks of the BK7 standard. We have included these details in the caption of the revised Fig. S5.

Overall, this is an interesting and potentially impactful study. I believe it is suitable for publication in Nature Communications after the authors have satisfactorily addressed the points raised above.

We thank the reviewer for the constructive comments and suggestions. We hope that the revised manuscript has clarified the issues raised and adequately addressed the reviewer's concerns.

References

Geballe, Z.M., Arveson, S.M., Speziale, S., Jeanloz, R., 2022. Sound speed and refractive index of amorphous CaSiO3 upon pressure cycling to 40 GPa. American Mineralogist 107, 2212–2218. https://doi.org/10.2138/am-2022-8081

Gréaux, S., Irifune, T., Higo, Y., Tange, Y., Arimoto, T., Liu, Z., Yamada, A., 2019. Sound velocity of CaSiO3 perovskite suggests the presence of basaltic crust in the Earth's lower mantle. Nature 565, 218–221. https://doi.org/10.1038/s41586-018-0816-5

Komabayashi, T., Hirose, K., Sata, N., Ohishi, Y., Dubrovinsky, L.S., 2007. Phase transition in CaSiO3 perovskite. Earth and Planetary Science Letters 260, 564–569. https://doi.org/10.1016/j.epsl.2007.06.015

Kudo, Y., Hirose, K., Murakami, M., Asahara, Y., Ozawa, H., Ohishi, Y., Hirao, N., 2012. Sound velocity measurements of CaSiO3 perovskite to 133 GPa and implications for lowermost mantle seismic anomalies. Earth and Planetary Science Letters 349–350, 1–7. https://doi.org/10.1016/j.epsl.2012.06.040

Murakami, M., Asahara, Y., Ohishi, Y., Hirao, N., Hirose, K., 2009. Development of *in situ* Brillouin spectroscopy at high pressure and high temperature with synchrotron radiation and infrared laser heating system: Application to the Earth's deep interior. Physics of the Earth and Planetary Interiors, Advances in High Pressure Mineral Physics: from Deep Mantle to the Core 174, 282–291. https://doi.org/10.1016/j.pepi.2008.07.030

Perks, N.J., Zhang, Z., Harrison, R.J., Carpenter, M.A., 2014. Strain relaxation mechanisms of elastic softening and twin wall freezing associated with structural phase transitions in (Ca,Sr)TiO3 perovskites. J. Phys.: Condens. Matter 26, 505402. https://doi.org/10.1088/0953-8984/26/50/505402

Salje, E.K.H., 2013. Elastic excitations in BaTiO3 single crystals and ceramics: Mobile domain boundaries and polar nanoregions observed by resonant ultrasonic spectroscopy. Phys. Rev. B 87. https://doi.org/10.1103/PhysRevB.87.014106

Stixrude, L., Lithgow-Bertelloni, C., Kiefer, B., Fumagalli, P., 2007. Phase stability and shear softening in CaSiO3 perovskite at high pressure. Phys. Rev. B 75, 024108. https://doi.org/10.1103/PhysRevB.75.024108

Thomson, A.R., Crichton, W.A., Brodholt, J.P., Wood, I.G., Siersch, N.C., Muir, J.M.R., Dobson, D.P., Hunt, S.A., 2019. Seismic velocities of CaSiO3 perovskite can explain LLSVPs in Earth's lower mantle. Nature 572, 643–647. https://doi.org/10.1038/s41586-019-1483-x
